# Supplementary material for: Transition metal-like carbocatalyst
Source: Nat Commun. 2020 Aug 14;11:4091. doi: 10.1038/s41467-020-17909-8 (PMC7427970; doi:10.1038/s41467-020-17909-8)
Supplement: Supplementary file 1 — Supplementary Information [file 41467_2020_17909_MOESM1_ESM.pdf]

Supplementary Information

# Transition Metal-Like Carbocatalyst

Luo et al.

## Supplementary Methods

**Chemicals.** Ethylenediamine (≥99.5%), propanediamine (99%), butanediamine (99%), carbon tetrachloride (≥99.5%), carbon tetrachloride (≥99.9%), melamine (AR), glucose (≥99.5%), P123 (Mn~5,800), TEOS (98%), 1-phenylethanol (99.5%), 2-phenylethanol (≥99%), ethylbenzene (≥99%), styrene (≥99%), phenylacetylene (≥98%), benzyl phenyl ether (≥98%), decane (≥99%), 1,2,3,4-tetrahydroquinoline (THQ, ≥98%), quinoline (%), $CuCl_2$ (97 %), $NiCl_2$ (98 %), guaiacol (≥98%), sodium carbonate (≥99%), formaldehyde (37% aq solution), guaiacol (>98%), and dodecane (≥99%) were purchased from Sigma-Aldrich. DMSO-$d_6$ (99.5%, 99.9 atom% D), benzene-$d_6$ (99.5%, 99.5 atom% D), $CDCl_3$-$d$ (99.5%, 99.8 atom% D), methanol-$d_4$ (99.5%, 99 atom% D), 2-PrOH-$d_8$ (99.5%, 99.8 atom% D), and 2-PrOH-$d_1$ (98 atom% D) were purchased from Cambridge Isotope Laboratories. Hydrochloric acid (35 – 38%, TraceMetal grade), hydrofluoric acid (48 – 51% solution in water, TraceMetal grade), nitric acid (67 – 71%, TraceMetal grade), methylene chloride (≥99.5%), methanol (99.8%), magnesium sulfate anhydrous (certified grade), sodium borohydride, (99%), nitrobenzene (99%), acetophenone (99%), and 2-PrOH (99.9%) were purchased from Fisher Chemical without further purification. All three inorganic acids are certified to contain less than <1 ppb Co, Cu, Fe, Ni, Ru, Pd, Pt, Rh, Al, Sb, As, Ba, Be, Bi, Cd, Ca, Ce, Cs, Cr, Dy, Er, Eu, Gd, Ga, Ge, Au, Hf, Ho, In, La, Pb, Li, Lu, Mg, Mn, Hg, Mo, Na, Nd, Nb, K, Pr, Re, Rb, Sm, Sc, Se, Ag, Na, Sr, Ta, Te, Tb, TI, Th, Tm, Sn, Ti, W, U, V, Yb, Y, Zn, and Zr. Ethylenediamine (≥99%), $FeCl_3$ (98 %), and mesitylene (99%) was purchased from Acros Organics. Allylbenzene (99%) was purchased from Alfa Aesar. Guaiacylglycerol-β-guaiacyl ether (≥97%) and 2-phenoxyacetophenone (98%) was purchased from TCI Chemical. 2-Phenoxy-1-phenylethan-1-ol (PPE, 99.9%) was purchased from BBLDpharm. 3',4'-Dimethoxyacetophenone (99%) was purchased from BBLDpharm. Pd/C (1 wt%) was purchased from Strem Chemicals. Carbon black (black pearls 2000) was purchased from Cabot Corporation. $H_2$ (99.995%) and $D_2$ (99.99%) were purchased from MATHESON trigas. All chemicals were used as received.

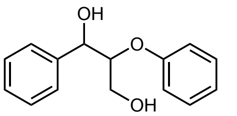


**Synthesis of 2-phenoxy-1-phenylpropane-1,3-diol (**PPDE, **3**): PPDE was prepared as follows[1,2]. An oven dried Schlenk flask was flushed with nitrogen and was charged with 2-phenoxyacetophenone (2.12 g, 10.0 mmol), $Na_2CO_3$ (1.27 g, 12.0 mmol), DMSO (5 mL) and EtOH (30 mL). The mixture was allowed to stir for 10 minutes and formaldehyde (37 % aq solution, 1.45 mL) was added. The resulting mixture was stirred at 50 C for 5 h. After that the mixture was cooled to room temperature and 90 mL of water was added. At this point dropwise HCl (3 M) was added until the pH reached 7. The suspension was extracted with $CH_2Cl_2$ (3 x 50 mL) and the organic part was dried over $MgSO_4$, filtered and the solvent was evaporated under vacuum to give an oil. The residue was mixed with dry MeOH (100 mL), followed by the addition of $NaBH_4$ (300 mg) at 0 ºC. The solution was allowed to stir at 25 ºC for 6 h and mixed with saturated aqueous $NH_4NO_3$ (120 mL). The mixture was extracted with $CH_2Cl_2$ (3 x 50 mL) and the organic phase was dried over $MgSO_4$, filtered and the solvent was evaporated under vacuum to give an oil, which was purified by silica gel chromatography (60% ethyl acetate in hexanes). Yield: 1.12 g (47 %). The product is a mixture of two diastereomers (74% vs 26%). $^1$H NMR (DMSO-$d_6$, 600 MHz): δ7.40 (m, 2H, *o*-$C_6H_5$CH), 7.36 (t, $^3J_{HH}$ = 7.8 Hz, 2H, *m*-$C_6H_5$CH), 7.29-7.17 (m, 3H, *p*-$C_6H_5$CH, *m*-$C_6H_5$O), 6.94-6.84 (m, 3H, *o*, *p*-$C_6H_5$O), 5.54-5.49 (d, 1H, $C_6H_5$CH(O*H*)-), 4.84-4.72, (m, 2H, -$CH_2$O*H*, $C_6H_5$C*H*(OH)-), 4.37 (m, 1H, -C*H*($CH_2$OH)O$C_6H_5$), 3.64, 3.28 (m, 1H, -C$H_2$OH). $^{13}$C NMR (DMSO-$d_6$, 150

MHz): δ 159.5, 159.2, 143, 142.8, 129.8, 129.7, 128.21, 128.18, 127.5, 127.4, 127.1, 120.9, 116.63, 116.4, 115.9, 83.5, 83.4, 72, 71.4, 60.4. NMR spectra are provided in the end of the Supplementary Information. IR (KBr, $cm^{-1}$): 3376, 3062, 3024, 2910, 1591, 1489, 1405, 1339, 1221, 1077, 1026, 888, 750. Anal. Calcd for $C_{15}H_{16}O_3$: C, 73.79; H, 6.55; O, 19.65. Found: C, 73.75; H, 6.60; O, 19.65. LC-MS (ESI): Calcd for ($C_{15}H_{16}O_3$) m/z 244.1; found 244.0.

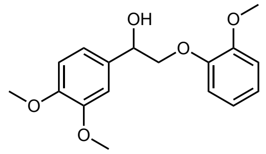


**Synthesis of 1-(3,4-Dimethoxyphenyl)-2-(2-methoxyphenoxy)ethan-1-ol (2)**: 1-(3,4-Dimethoxyphenyl)-2-(2-methoxyphenoxy)ethan-1-ol was prepared as described for PPDE (**3**) using 3',4'-dimethoxyacetophenone and guaiacol as reactants[1,2]. $^{1}$H NMR ($CDCl_3$, 600 MHz): δ3.91 (s, 3H), 3.92 (s, 3H), 3.93 (s, 3H), 4.00 (t, $J$ = 9.6 Hz, 1H), 4.21 (dd, $J_1$ = 9.9 Hz, $J_2$ = 3.0 Hz, 1H), 5.09 (dd, $J_1$ = 9.9 Hz, $J_2$ = 3.0 Hz, 1H), 6.88-7.05 (m, 7H). $^{13}$C NMR (DMSO-$d_6$, 600 MHz): δ 150.18, 149.13, 148.80, 148.01, 132.14, 122.61, 121.12, 118.64, 116.12, 112.02, 111.07, 109.42, 77.24, 77.03, 76.81, 76.43, 72.14, 55.97, 55.91. NMR spectra are provided in the end of the Supplementary Information. Anal. Calcd for $C_{17}H_{20}O_5$: C, 67.09; H, 6.62; O, 26.28. Found: C, 67.11; H, 6.54; O, 26.35.

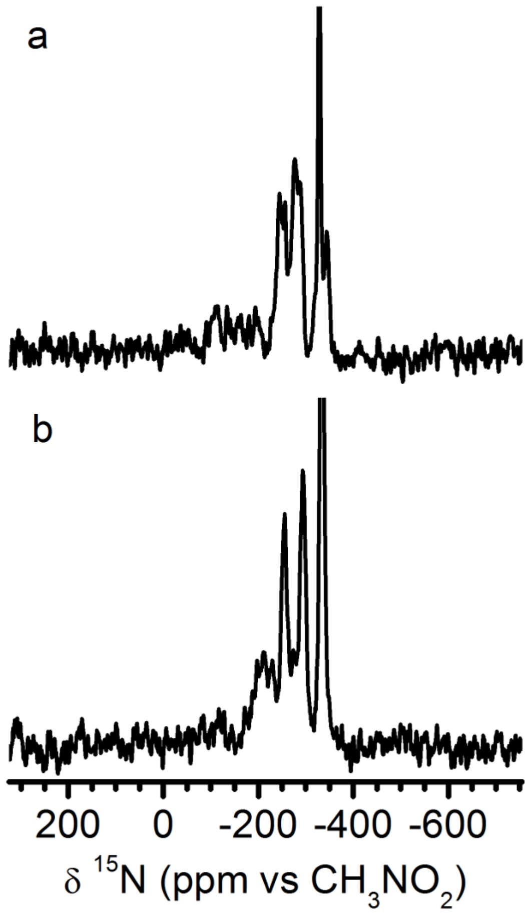


**Supplementary Fig. 1** DNP-enhanced $^{15}N\{^1H\}$ CPMAS NMR spectra of condensed polymer (a) prior to calcination and (b) after calcination for 10 min at 300 °C, recorded with a large spectral width.

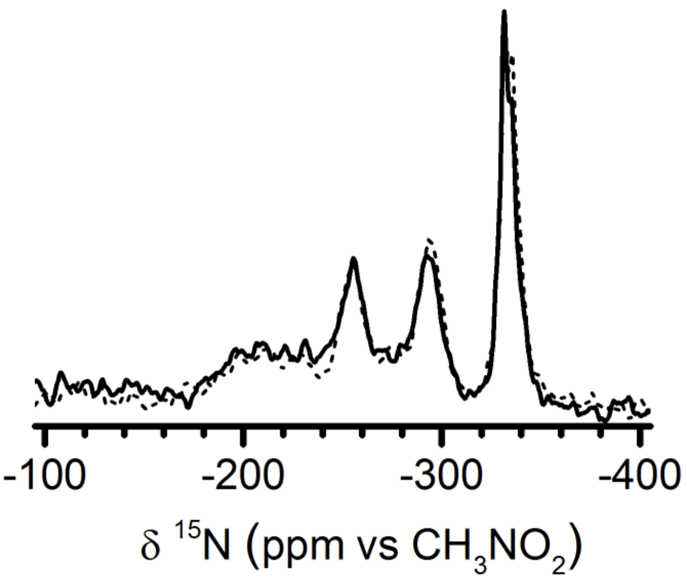


**Supplementary Fig. 2** DNP-enhanced $^{15}N\{^1H\}$ CPMAS NMR spectra taken with 4 ms (solid) and 2 ms (dashed) of CP contact time. In an earlier study of nitrogen-doped carbon materials[3], a similar $^{15}N$ signal was found and assigned to N-substituted pyrrole and imidazolium. In our experiment, the signal intensity is saturated after 2 ms of CP time, which suggests the signal is attributed to protonated nitrogens, i.e. in imidazolium rather than N-substituted pyrrole.

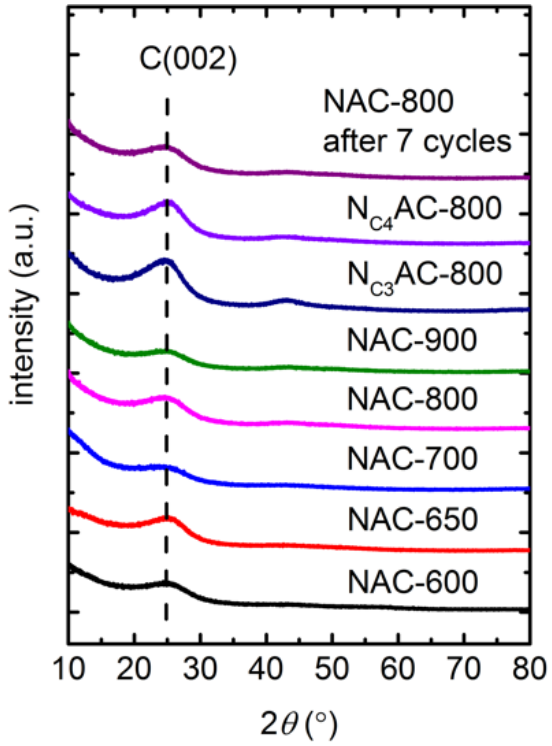


**Supplementary Fig. 3** Powder XRD patterns of NAC catalysts, as well as the NAC-800 after 7 reaction cycles, which show only the C (002) plane in graphitized carbons.

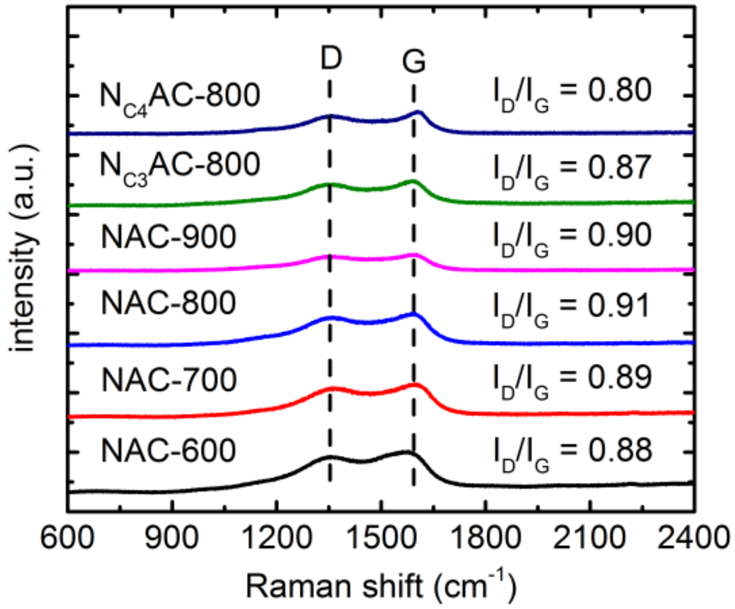


**Supplementary Fig. 4** Raman spectra of different NACs. The relative intensity of the D band compared with the G band gives a quantitative indication of the defect density of NAC catalysts.

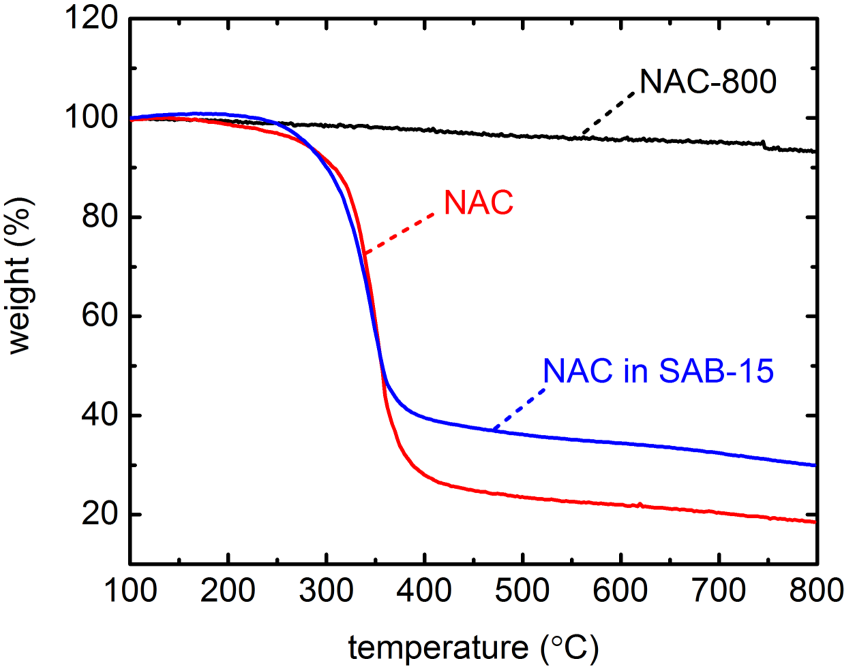


**Supplementary Fig. 5** Thermogravimetric analysis (TGA) of NAC-800, and condensed polymer of ethylenediamine and carbon tetrachloride with or without SBA-15 (at 90 °C). Less than 6% of mass loss was observed for NAC-800. The mass loss for condensed polymer with or without SBA-15 appears in the range of 250 to 450 °C.

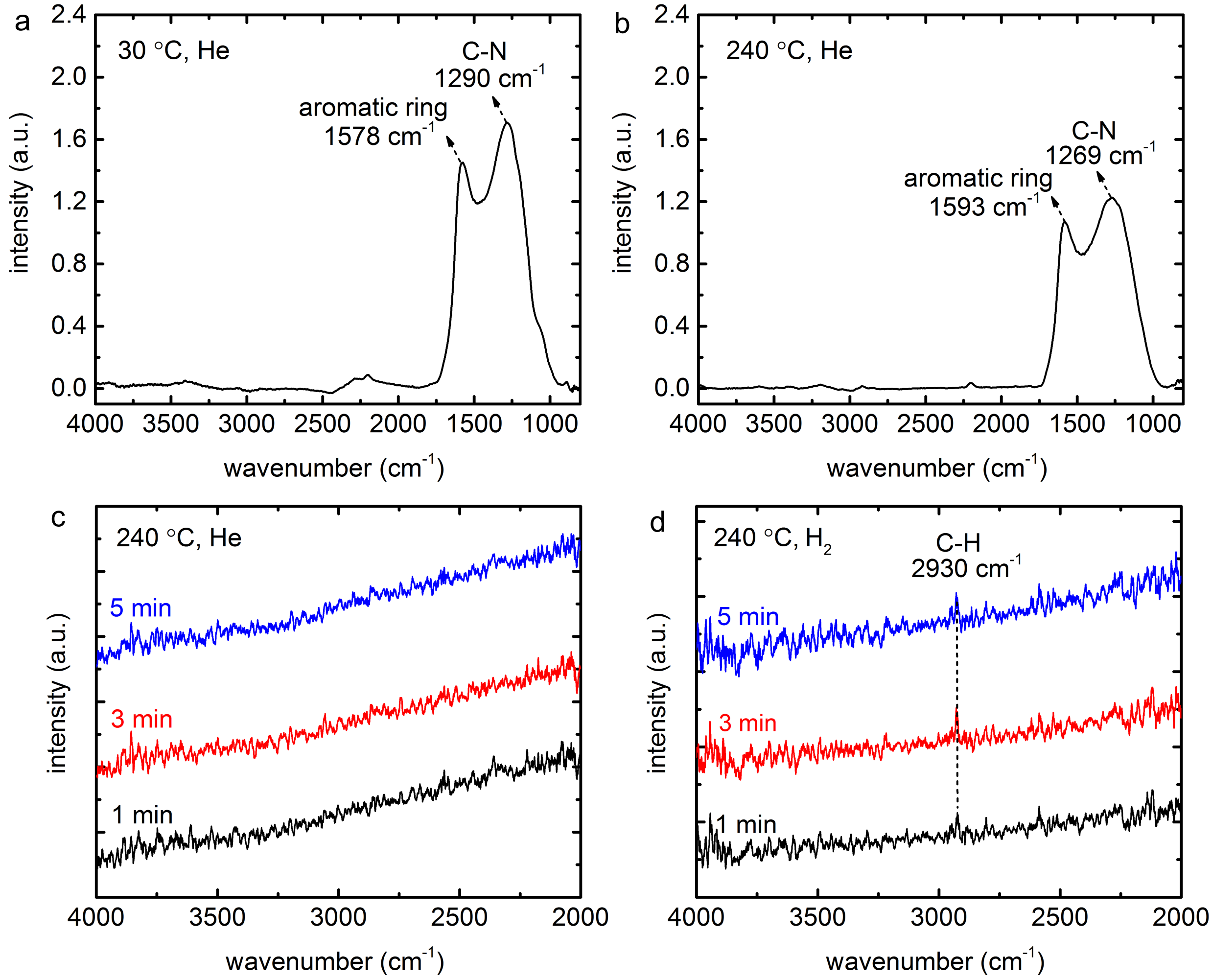


**Supplementary Fig. 6** The *in situ* DRIFTS spectra of NAC-800 (diluted 20× with KBr). (a) and (b) are collected under He at 30 and 240 °C, respectively, using the spectra of KBr under He at the corresponding temperature as the background. (c) and (d) are the time-resolved spectra at 240 °C under flowing He and $H_2$, respectively, using the spectrum of NAC-800 (diluted 20× with KBr) under He at 240 °C as the background. For (c) and (d), time was recorded either after reaching the desired temperature (for He) or after switching gas (for $H_2$). The DRIFTS spectrum of NAC-800 under the flow of He at 30 °C is primarily composed of graphitic $sp^2$ domains (1578-1593 $cm^{-1}$) and C−N bond (1269-1290 $cm^{-1}$)[4]. No change to the spectra was observed after increasing the temperature to 240 °C. *In situ* DRIFTS study shows the formation of C-H bond (at ~2930 $cm^{-1}$)[5] rather than O-H or N-H at 240 °C under the flow of $H_2$.[4]

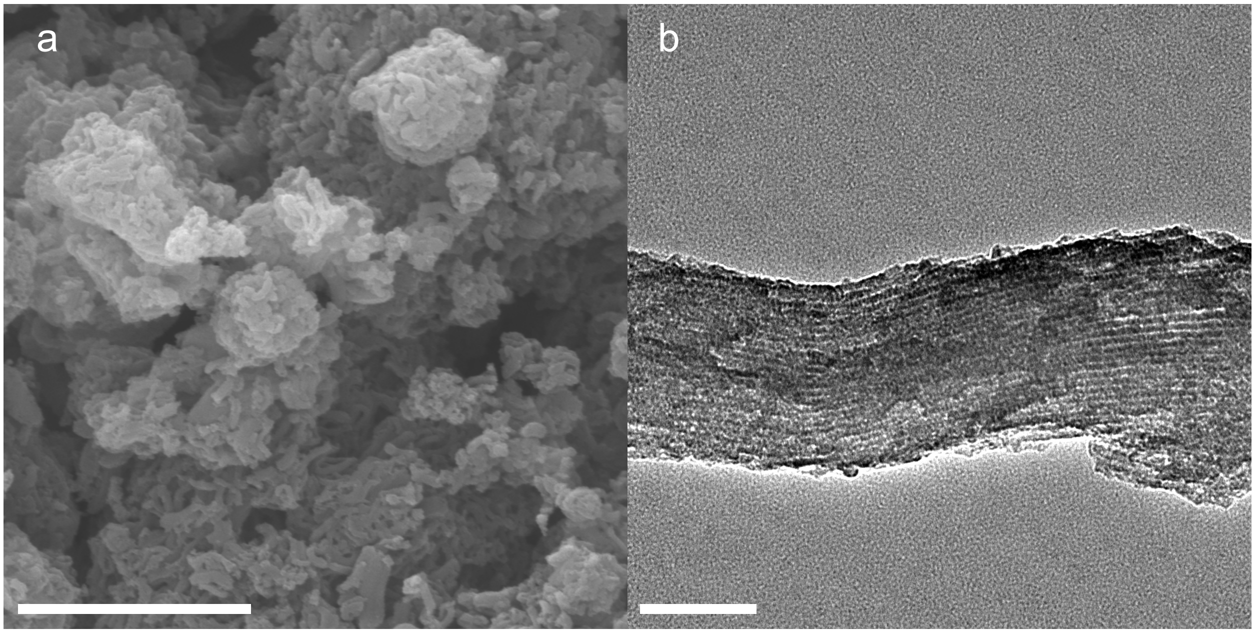


**Supplementary Fig. 7** Microscopic study of NAC-800 catalyst. a. The SEM image shows the rod-like morphology of NAC-800, analogous to the parent SBA-15 template. Scale bar, 2 um. b. The TEM image shows that the mesoporous channels in NAC-800 are periodically aligned over a large domain. Scale bar, 100 nm.

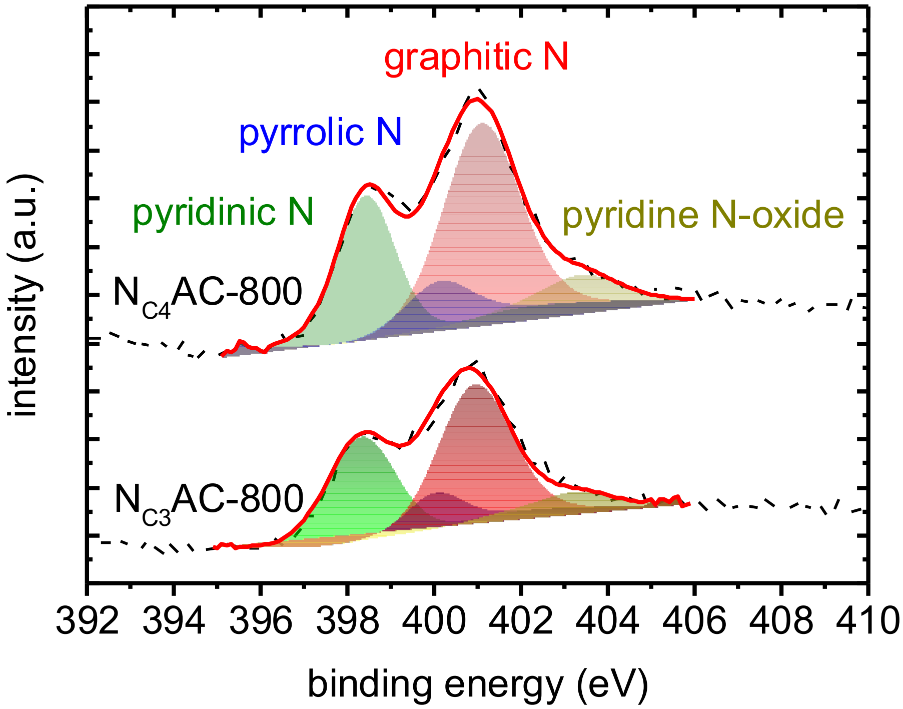


**Supplementary Fig. 8** N1*s* XPS spectra of NACs synthesized with propanediamine or butanediamine.

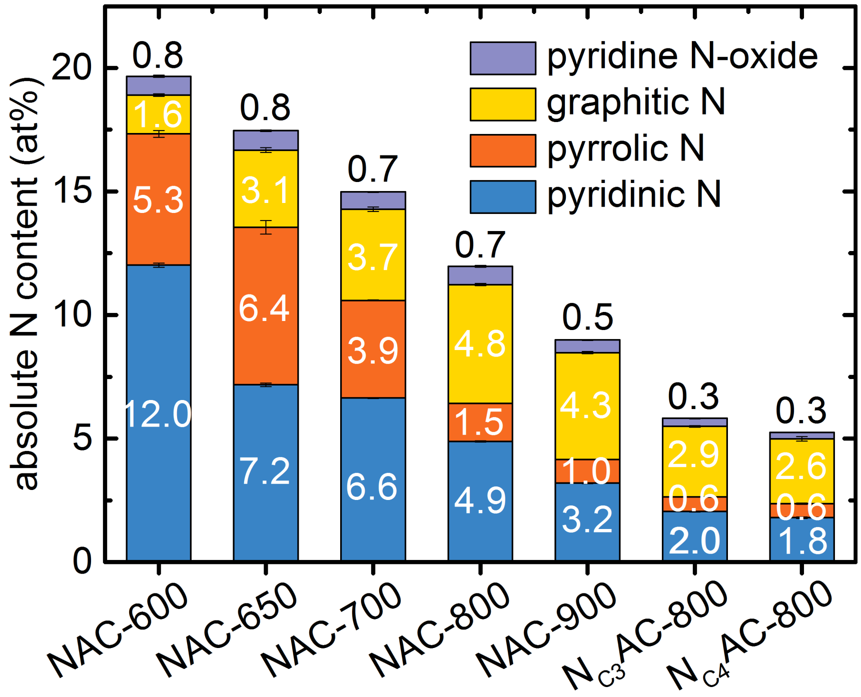


**Supplementary Fig. 9** The absolute contents of pyridinic, pyrrolic, graphitic, and pyridine N-oxide nitrogen species in NACs by deconvolution of XPS spectra. Higher calcination temperature leads to the decrease of both pyridinic and pyrrolic N, while graphitic N increases with temperature first at 600-800 °C and then drop at 900 °C. The total N contents of NACs synthesized with longer-chain diamines are lower. The error bars of absolute content of graphitic N were estimated and given in Supplementary Table 3.

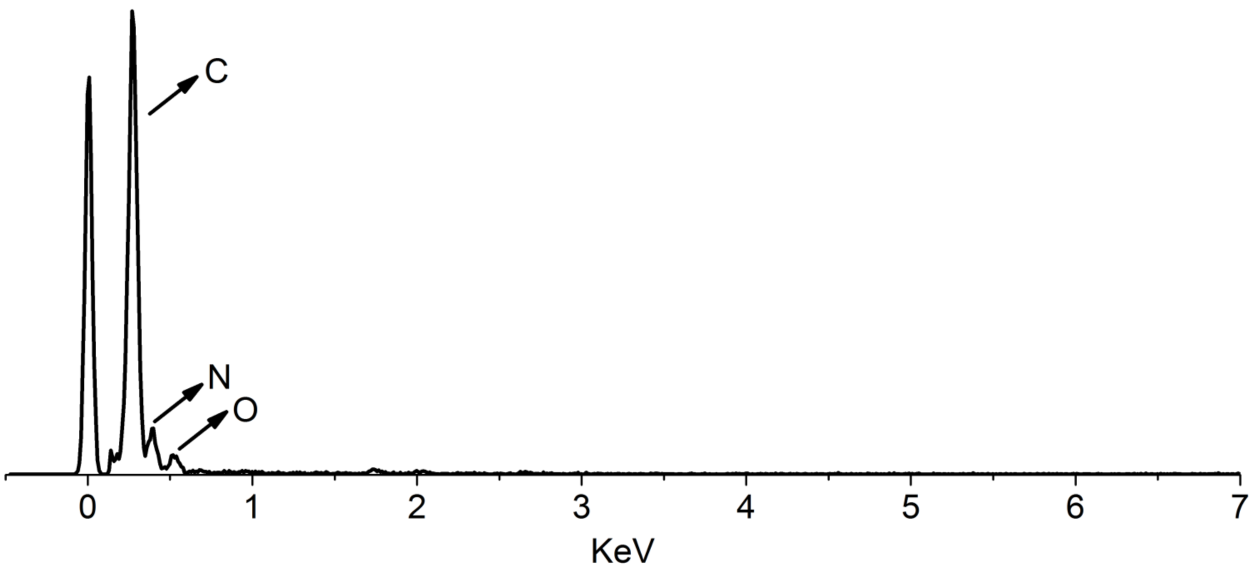


**Supplementary Fig. 10** STEM-EDX spectrum of NAC-800. Apart from the C, N, O species, no other metal impurities were detected. The O species is partially contributed by possible oxygen impurities from the water in the air or adsorbed on the catalyst surface.

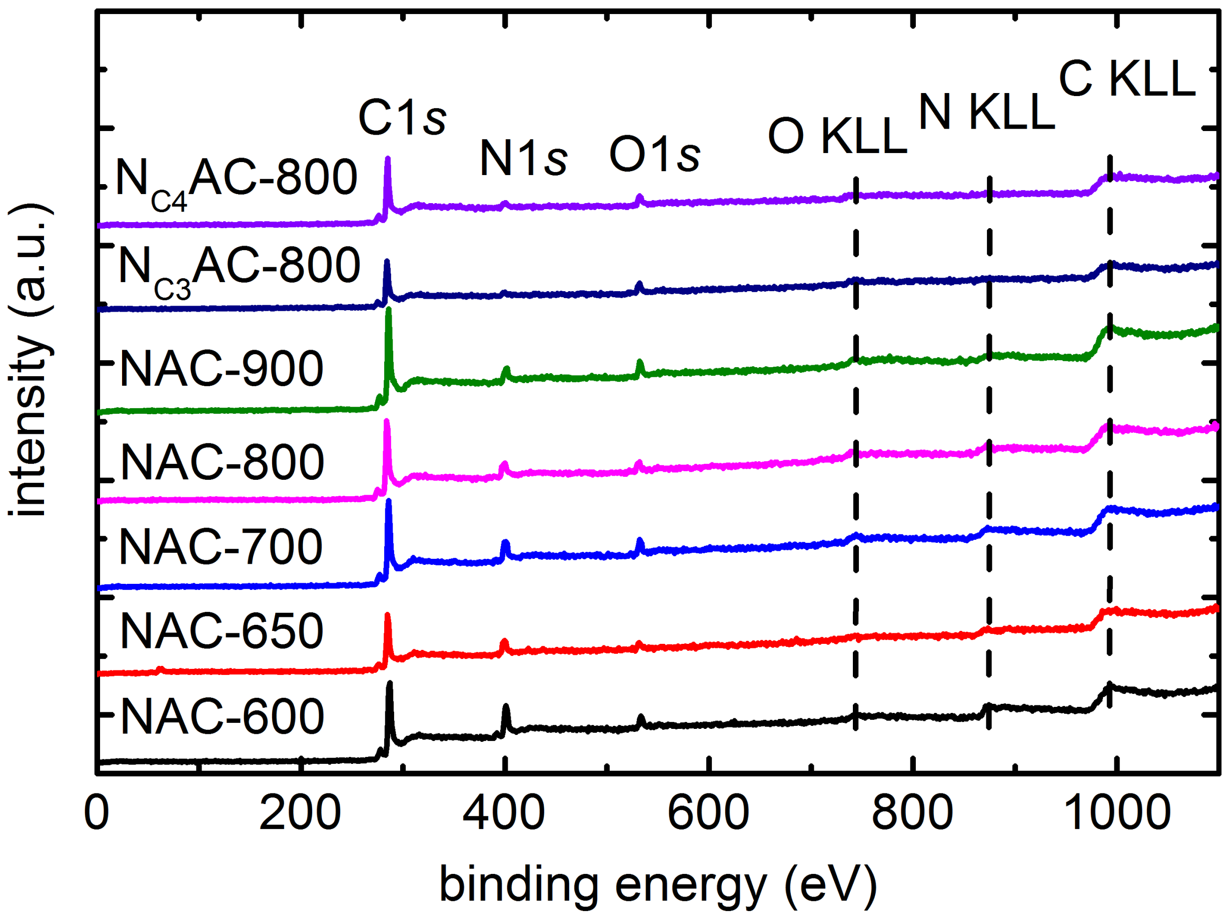


**Supplementary Fig. 11** XPS wide-scan spectra of different NACs, showing no metal impurities except C, N and O signals.

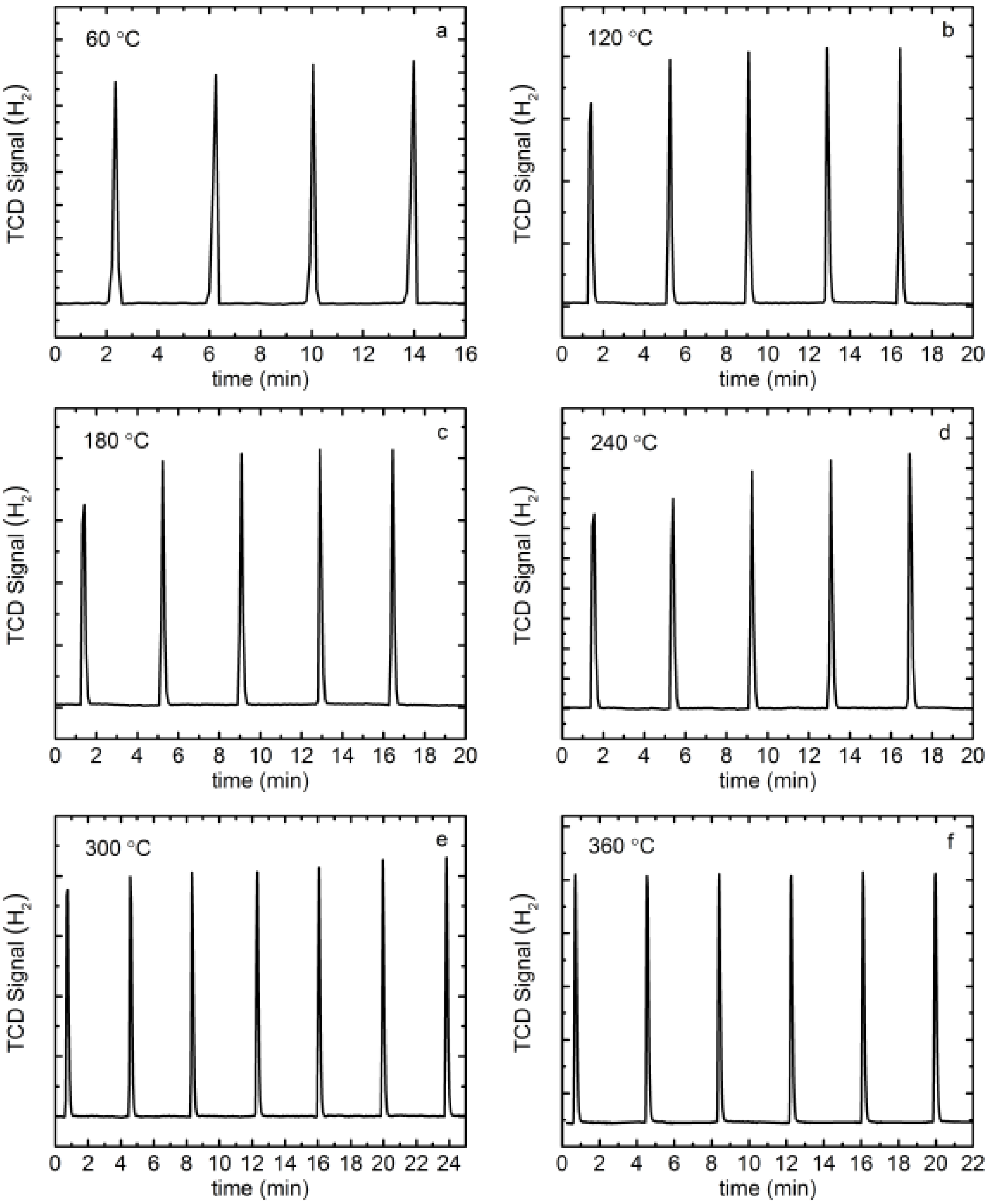


**Supplementary Fig. 12** $H_2$ pulsed chemisorption of NAC-800 at 60-360 °C.

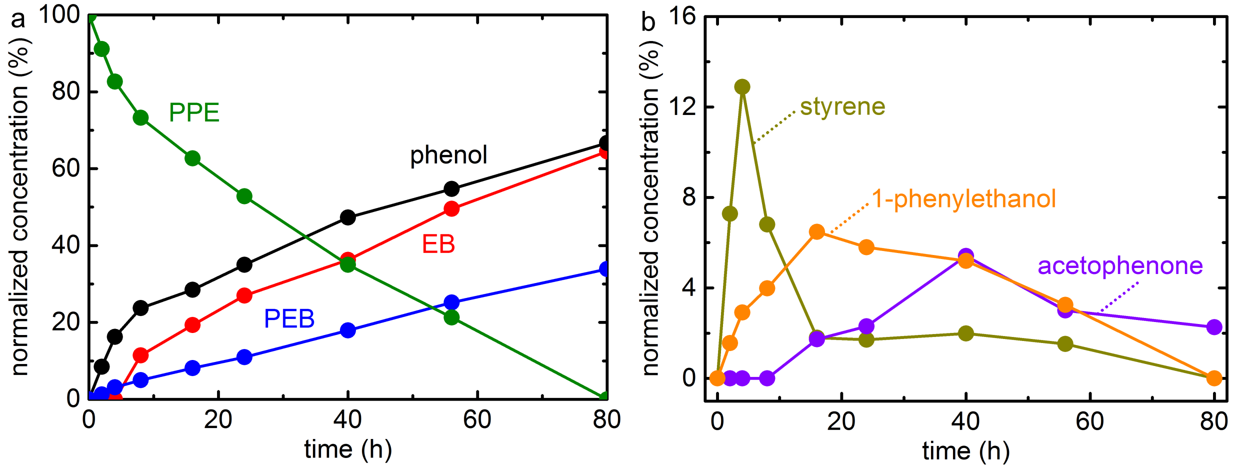


**Supplementary Fig. 13** Time plots of species concentrations of (a) reactant and products, and (b) intermediates for PPE conversion over NAC-800 catalyst. Reaction conditions: 14 mmol $L^{-1}$ PPE in 2-PrOH (1.50 mL), NAC-800 (5.0 mg), 230 °C, 20 bar $H_2$.

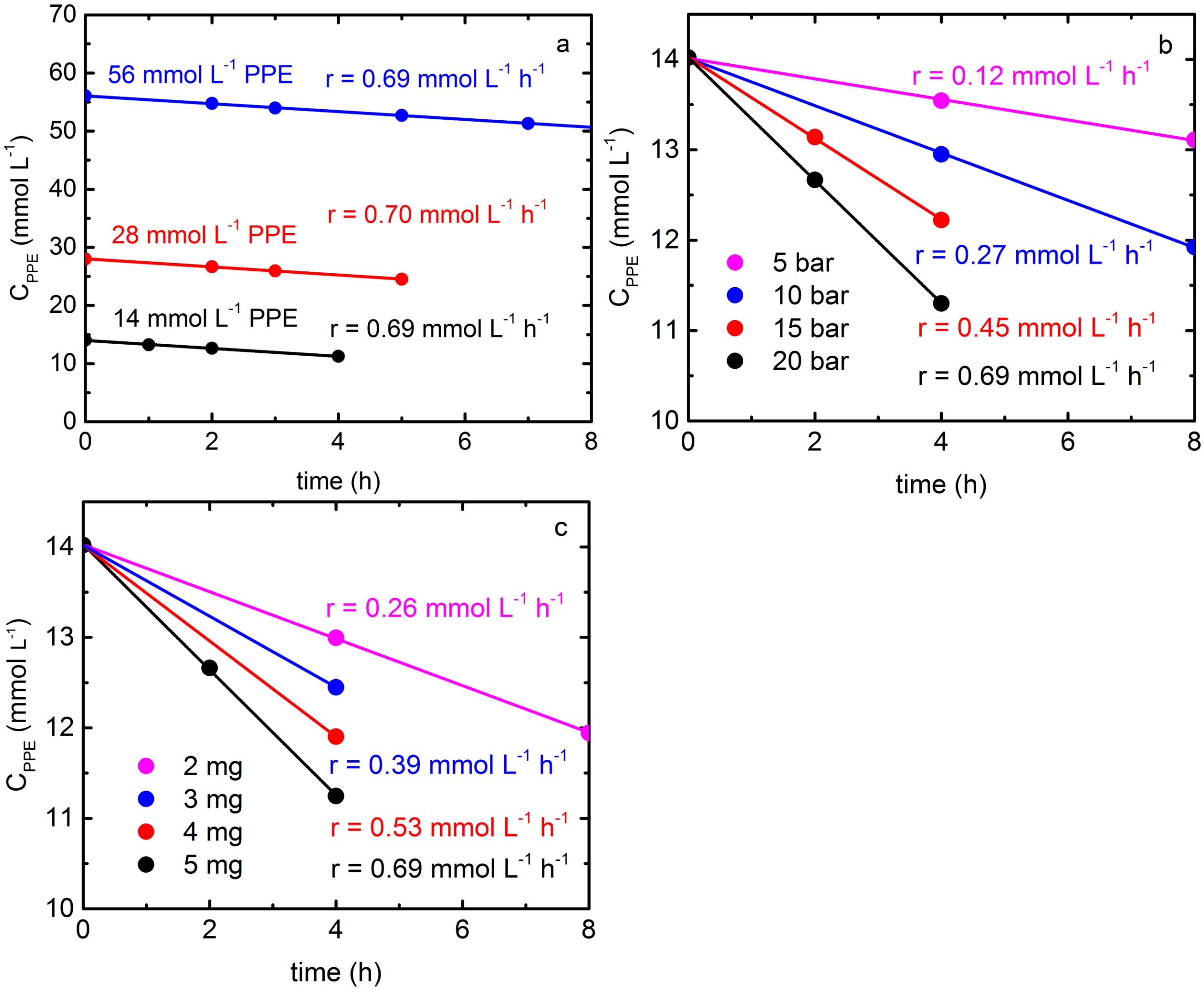


**Supplementary Fig. 14** PPE concentrations at 230 °C versus time as functions of (a) PPE concentration (20 bar $H_2$), (b) $H_2$ pressure (14 mmol $L^{-1}$ PPE), and (c) catalyst amount (14 mmol $L^{-1}$ PPE, 20 bar $H_2$).

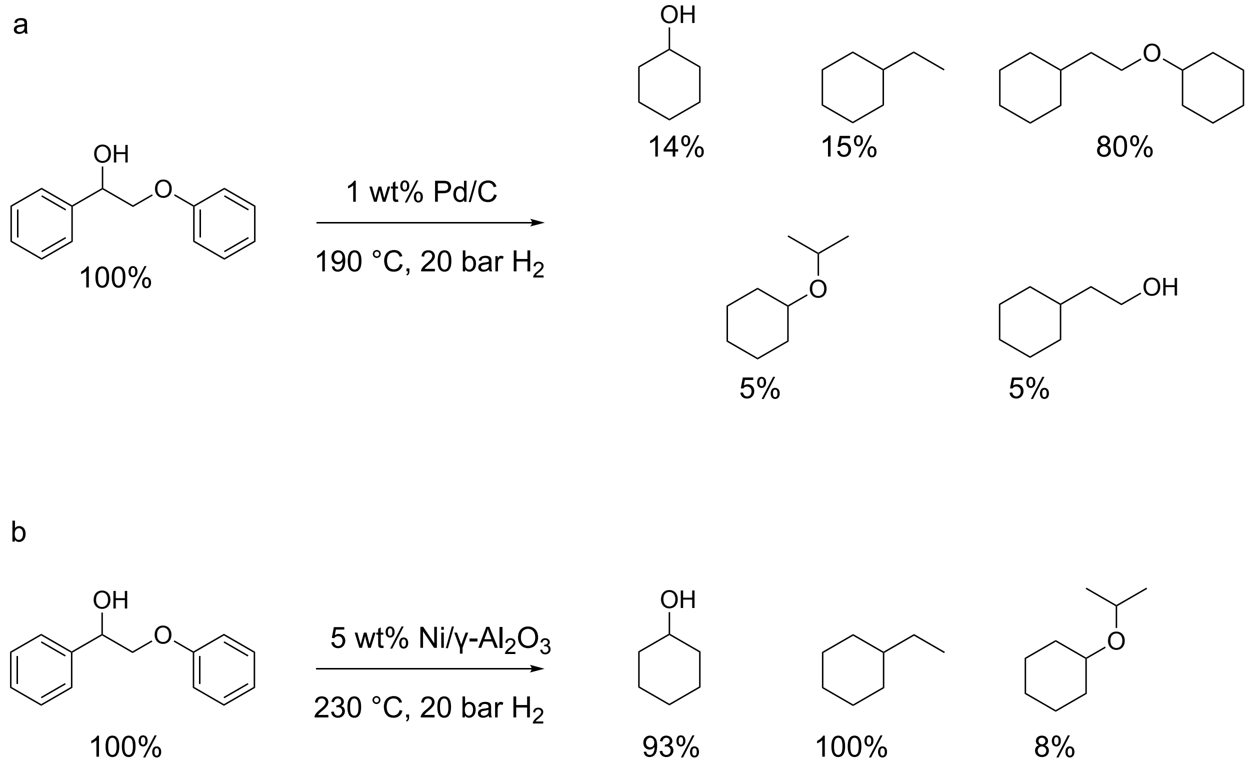


**Supplementary Fig. 15** Conversion of PPE by 1 wt% Pd/C and 5 wt% Ni/γ-$Al_2O_3$ catalysts. Conversion and yield are shown below PPE and corresponding products, respectively. Reaction conditions: (a) 1 wt% Pd/C (5.0 mg), 14 mmol $L^{-1}$ PPE in 2-PrOH (1.50 mL), $H_2$ (20 bar), 190 °C, 8 h. (b) 5 wt% Ni/γ-$Al_2O_3$ (5.0 mg), 14 mmol $L^{-1}$ PPE in 2-PrOH (1.50 mL), $H_2$ (20 bar), 230 °C, 16 h.

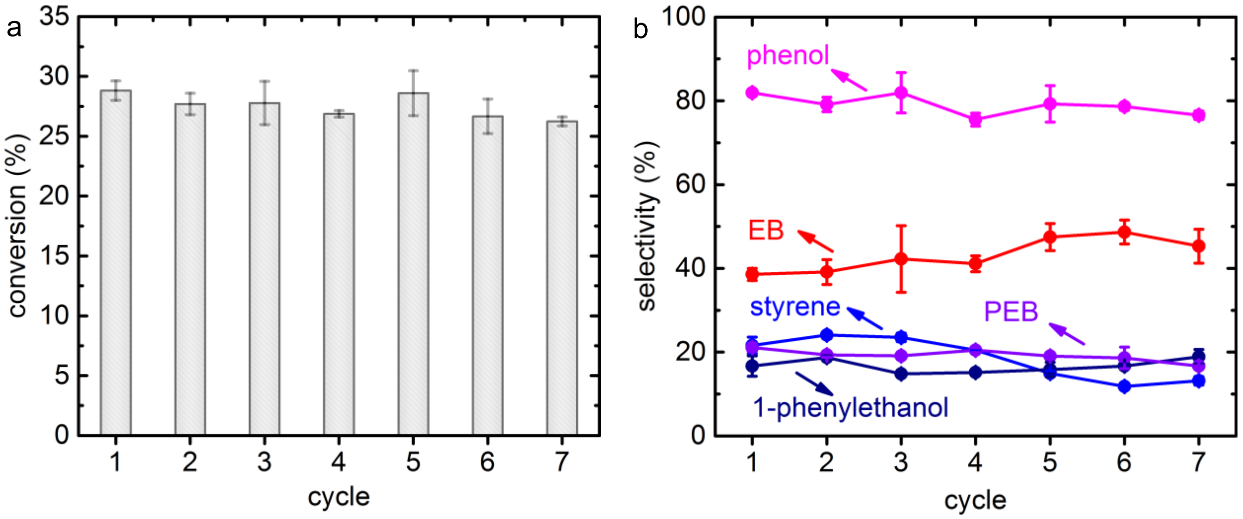


**Supplementary Fig. 16** Recycling study of NAC-800 for PPE conversion, showing (a) conversion of PPE, and (b) selectivity towards intermediates and products. Reaction conditions: NAC-800 (5.0 mg), 14 mmol $L^{-1}$ PPE in 2-PrOH (1.50 mL), $H_2$ (20 bar), 230 °C, 8 h.

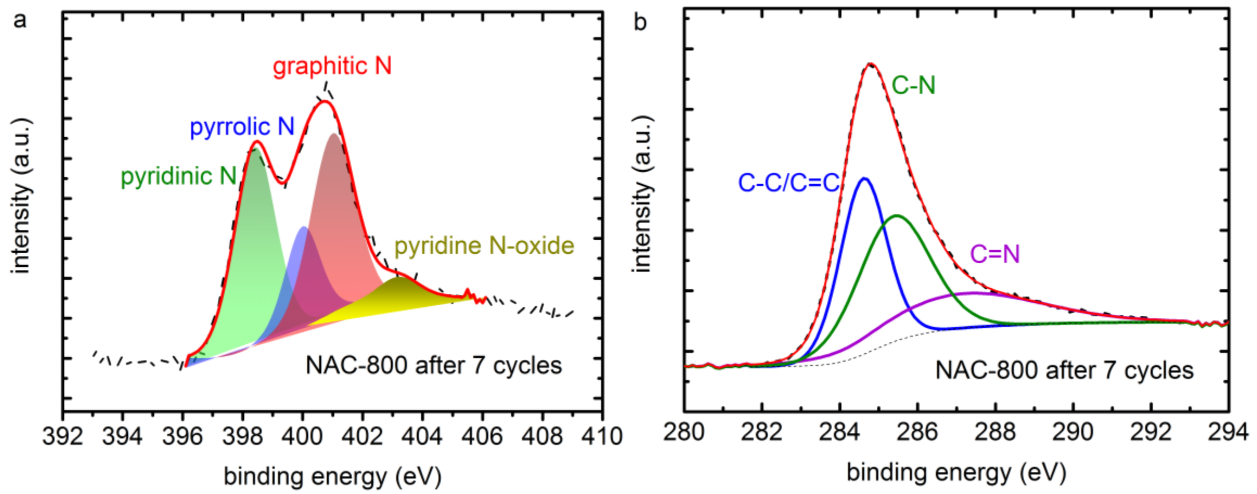


**Supplementary Fig. 17** N1*s* (a) and C1*s* (b) XPS spectra of NAC-800 after 7 reaction cycles.

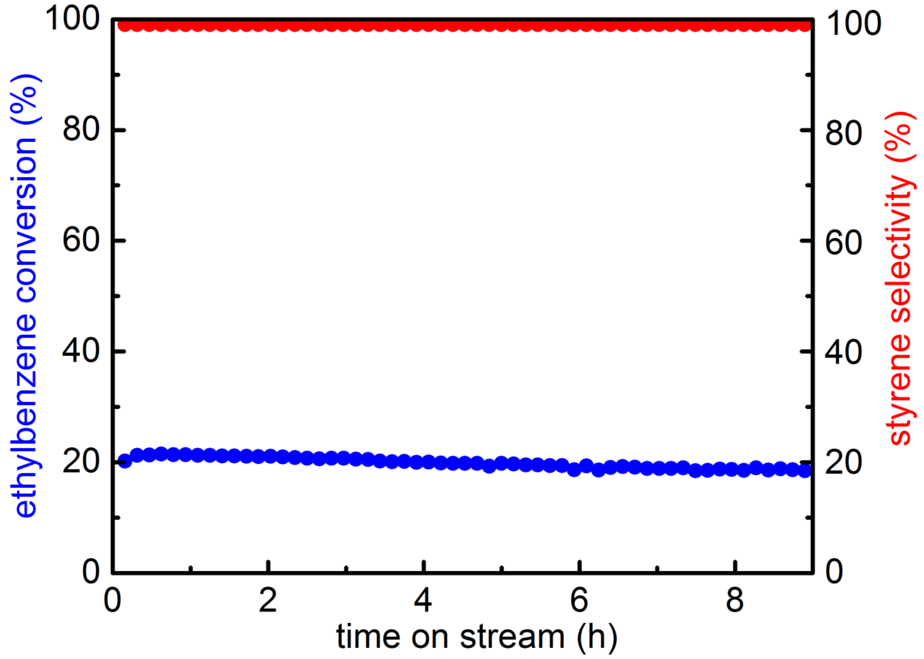


**Supplementary Fig. 18** A time-on-stream study of catalytic non-oxidative dehydrogenation of ethylbenzene to styrene at 550 °C. Reaction conditions: 20.0 mg NAC-800, 0.034 mL $min^{-1}$ ethylbenzene, 1.0 mL $min^{-1}$ $H_2$, balance with He to 50 mL $min^{-1}$ in total gas flow.

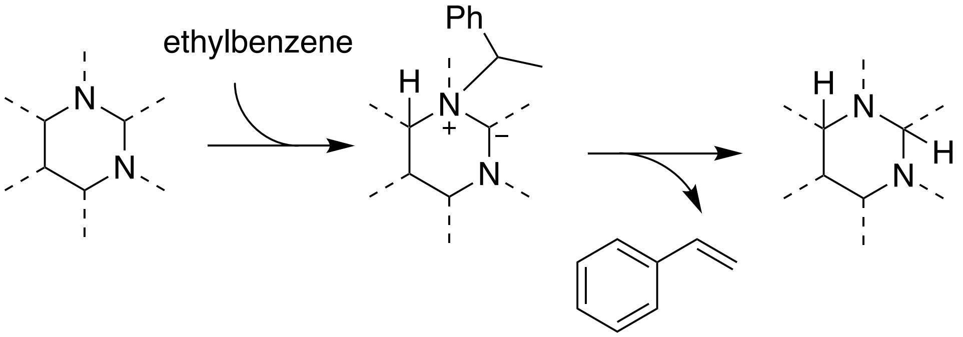


**Supplementary Fig. 19** Proposed mechanism for dehydrogenation of ethylbenzene to styrene over NAC-800.

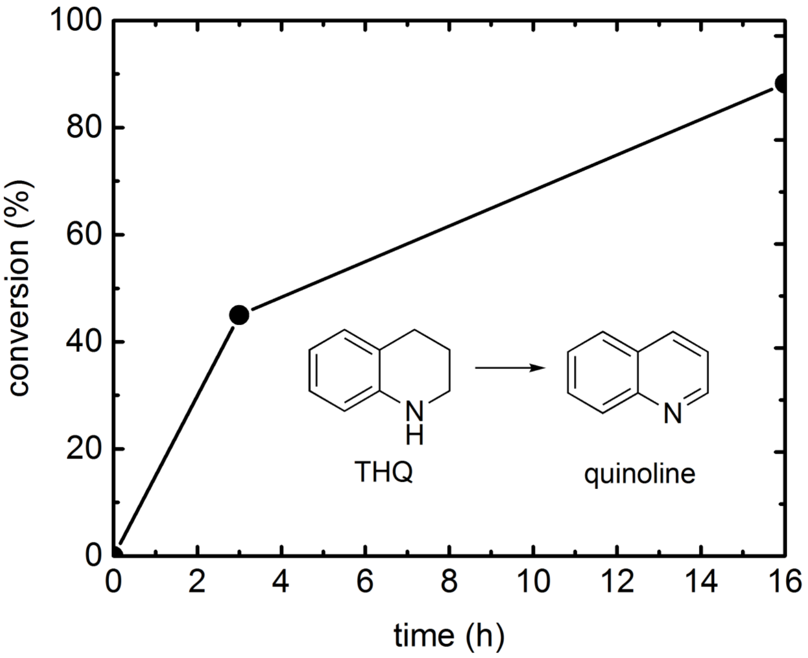


**Supplementary Fig. 20** Metal-free dehydrogenation of THQ over NAC-800 catalyst. Reaction conditions: NAC-800 (5.0 mg), THQ (0.100 mmol), mesitylene (2.00 mL), Ar (1 bar), 150 °C. The selectivities at both time points were 100%.

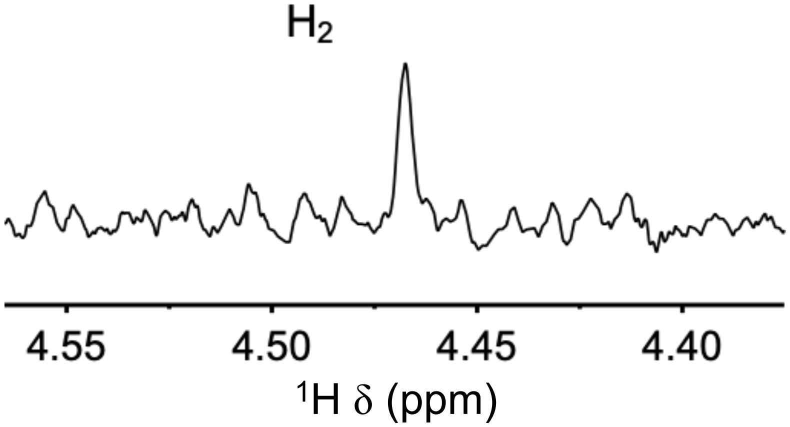


**Supplementary Fig. 21** $^1H$ NMR of benzene-$d_6$-dissolved $H_2$ from catalytic non-oxidative dehydrogenation of THQ over NAC-800. Reaction conditions: NAC-800 (5.0 mg), THQ (0.100 mmol), mesitylene (2.00 mL), $N_2$ (10 bar), 140 °C, 3 h.

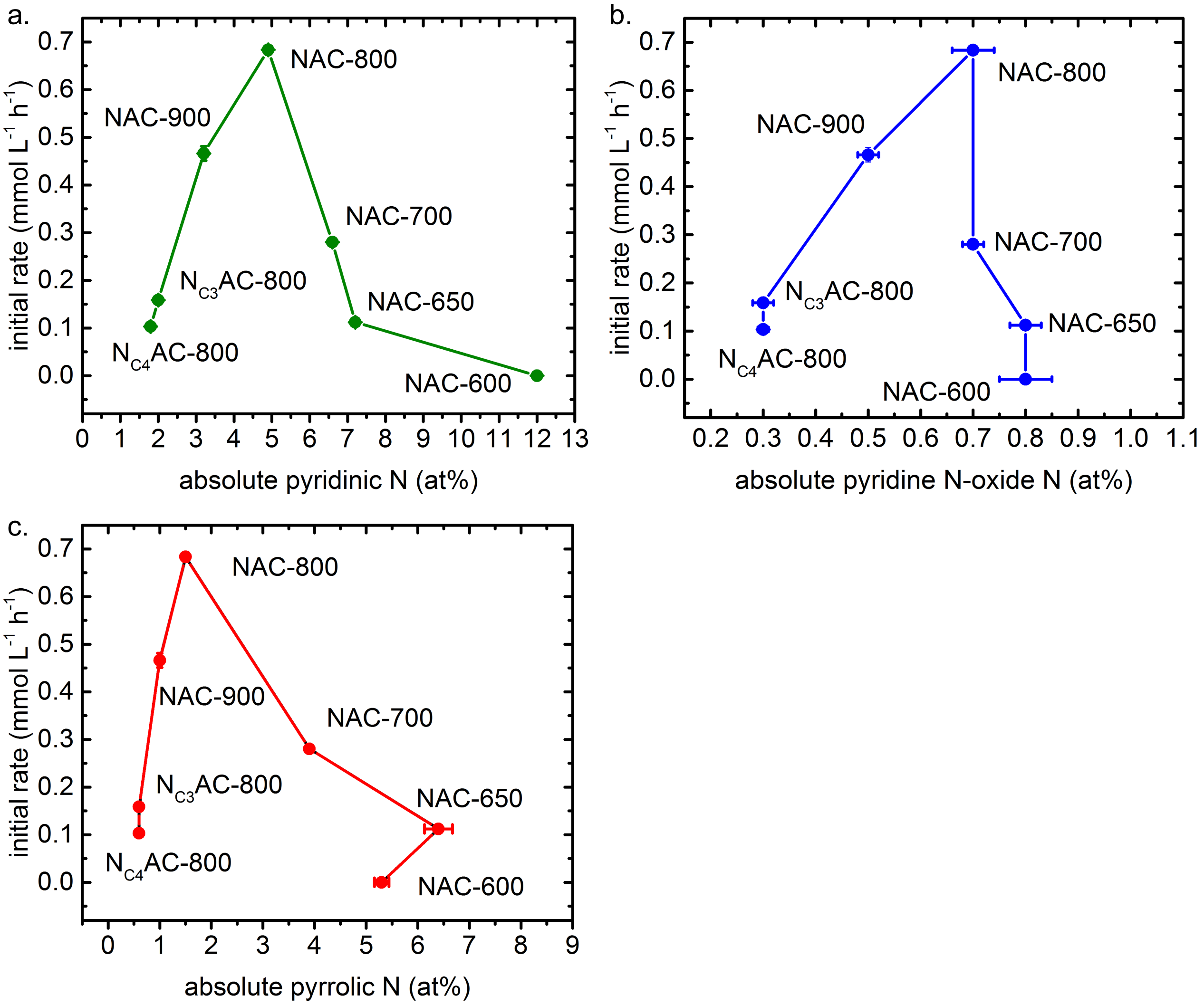


**Supplementary Fig. 22** The correlation between absolute contents of (a) pyridinic N, (b) pyridine N-oxide N, and (c) pyrrolic N of different NAC catalysts and corresponding initial rates of PPE conversions. No correlation of initial rates was found with N species except for graphitic N (Fig. 2d). The error bars of absolute N content were estimated and given in Supplementary Table 3 and the s.d. of the initial rates are approx. 3% of the mean values, obtained after repeated runs.

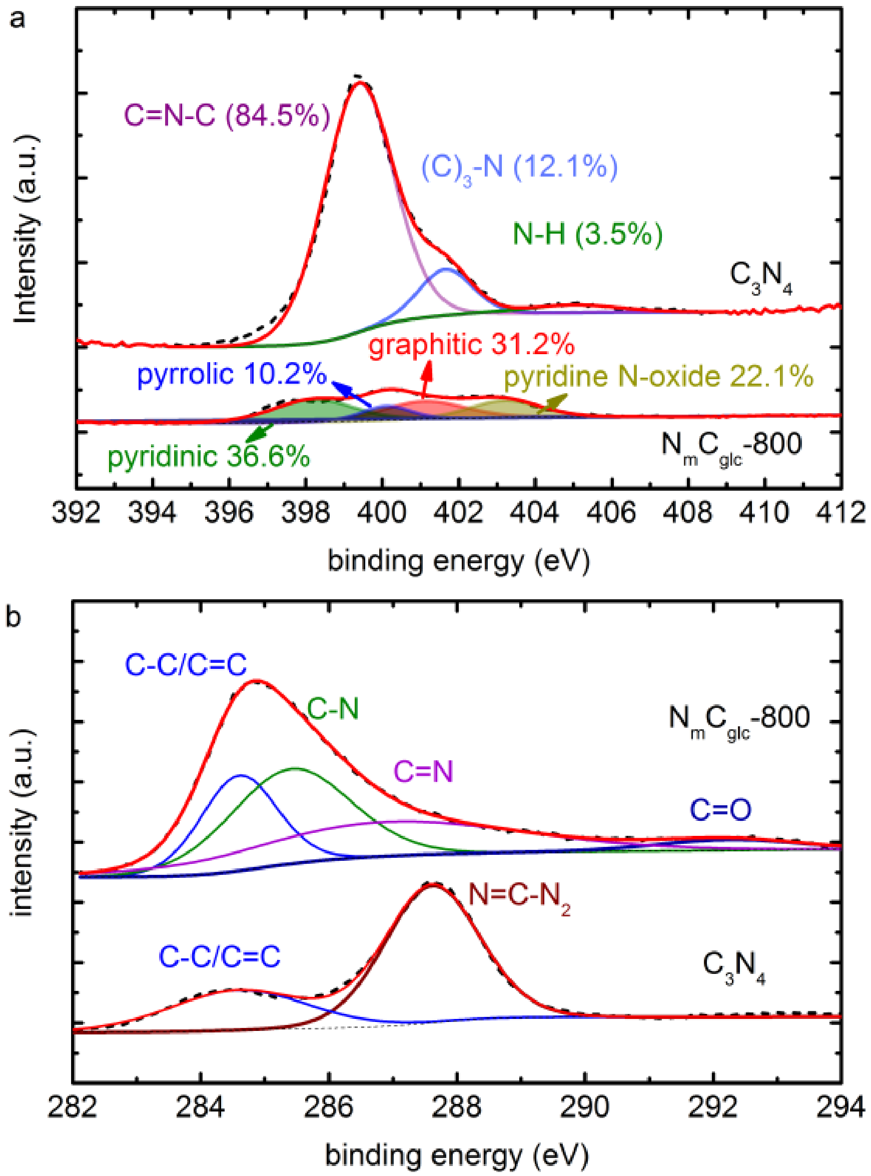


**Supplementary Fig. 23** (a) N1*s* and (b) C1*s* XPS spectra of $C_3N_4$ and $N_mC_{glc}$-800. The N in $C_3N_4$ mainly existed as tri-s-triazine species, while the Ns in $N_mC_{glc}$-800 exist as four regular type of N species.

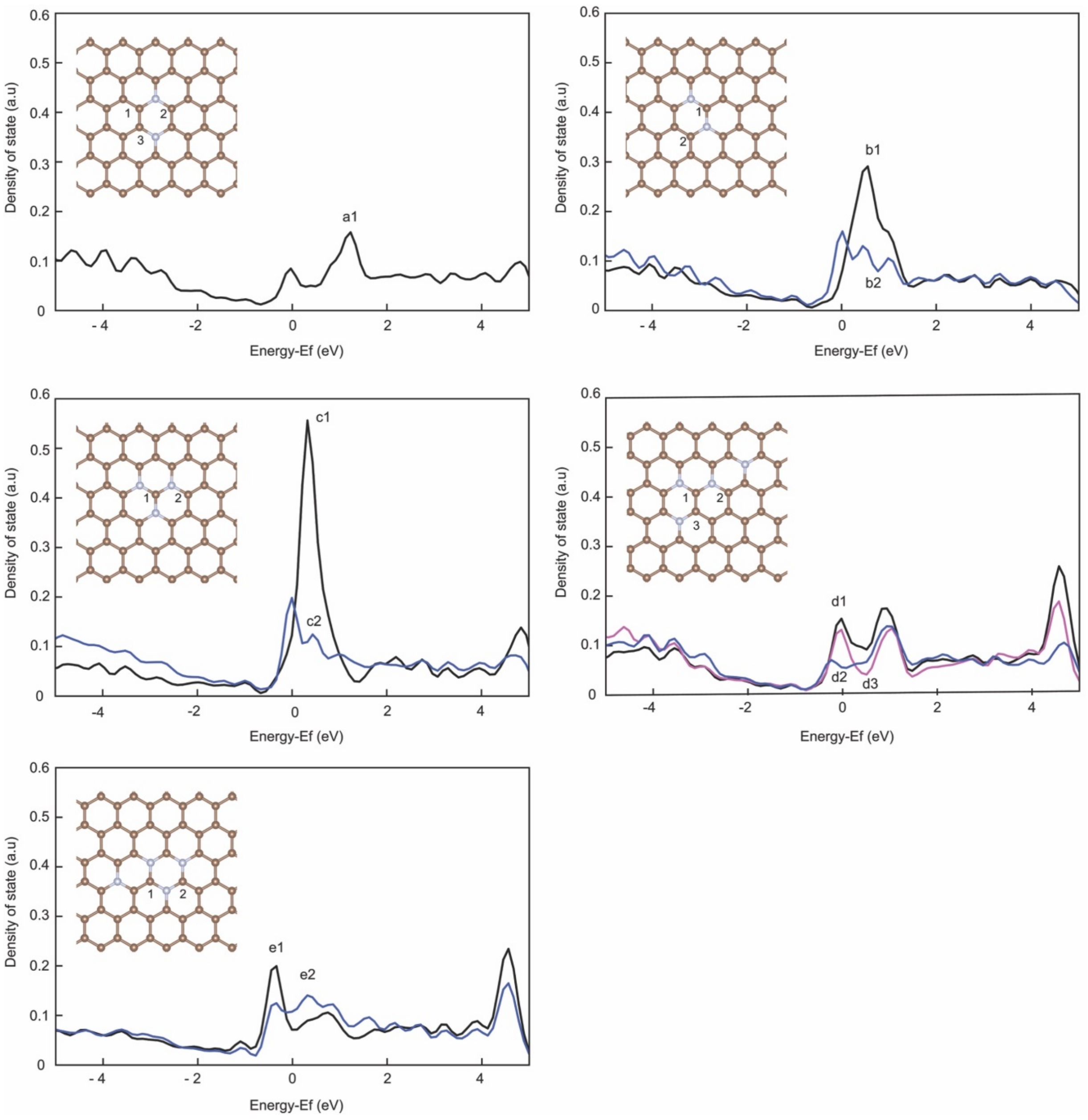


**Supplementary Fig. 24** DFT calculated projected density of states onto carbon adjacent to the N dopants. The label is consistent with Fig. 4 in the main text. The atomic structures are shown together with the DOS plot.

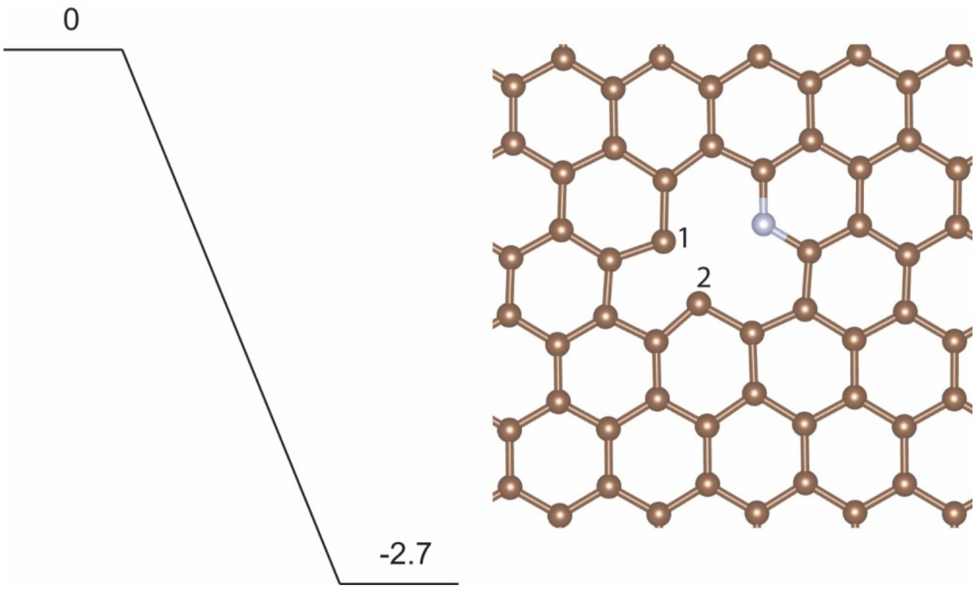


**Supplementary Fig. 25** DFT calculated barrierless dissociative adsorption of $H_2$ onto the two carbons labeled as 1 and 2 in the proximity of the pyridinic N. The large energy gain for $H_2$ dissociation prohibits further activity of the hydrogen atoms due to the high stability.

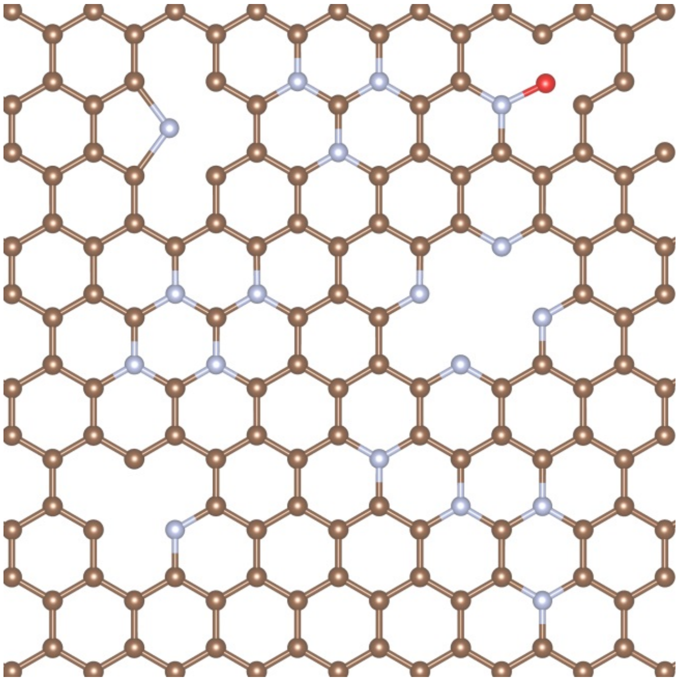

**Supplementary Fig. 26** Model of a high concentration of substitutional N in a graphene sheet.

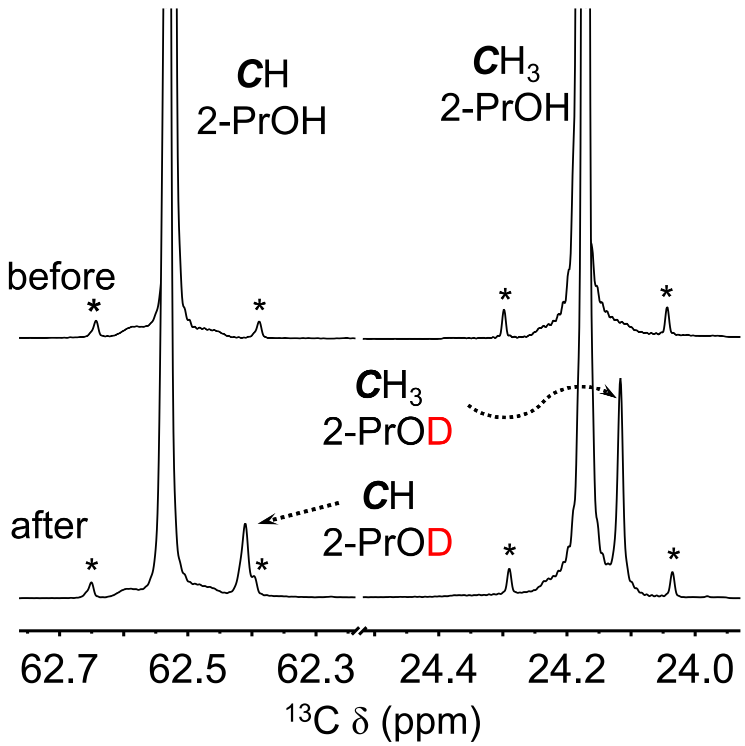


**Supplementary Fig. 27** $^{13}$C NMR spectra before and after isotropic exchange of O***H*** in 2-PrOH with ***D***$_2$ over NAC-800. $^{13}$C Satellite peaks are denoted as asterisk. Reaction conditions: NAC-800 (5 mg), 2-PrOH (1.5 mL), 230 °C, 20 bar $D_2$, 48 h.

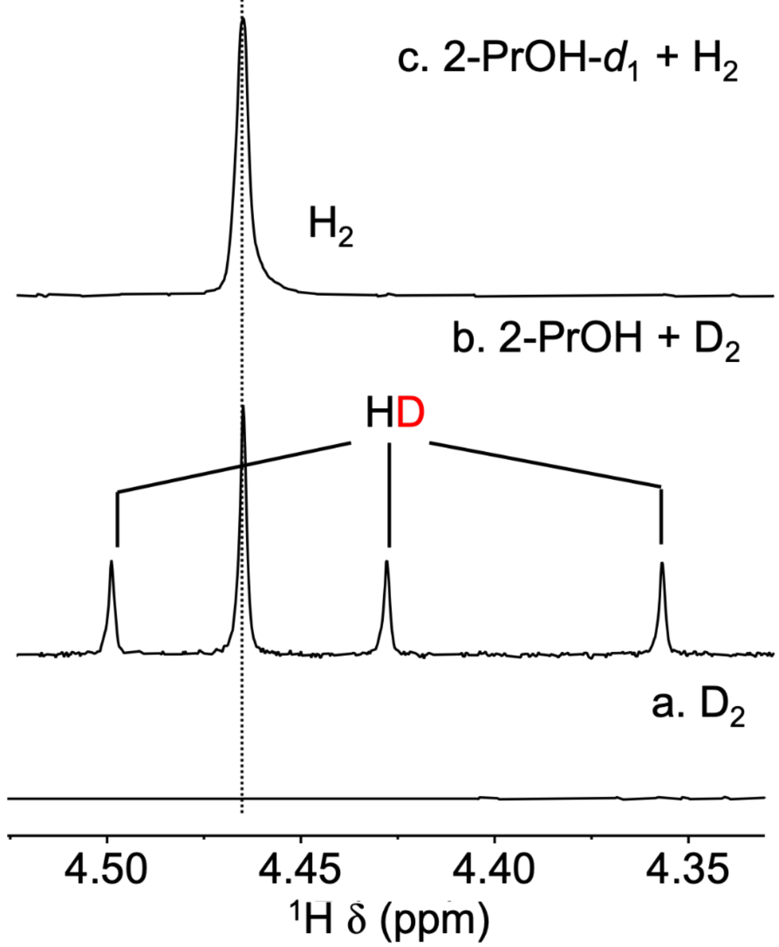


**Supplementary Fig. 28** $^1$H NMR spectra of the benzene-$d_6$ with dissolved gas: (a) pure $D_2$, showing no residual $H_2$ or HD was found in the starting $D_2$; (b) the headspace after the exchange reaction of 2-PrOH with $D_2$, showing the formation of HD and $H_2$; and (c) the headspace after the exchange reaction of 2-PrOH-$d_1$ with $H_2$ in the presence of PPE (14 mmol $L^{-1}$), showing only $H_2$. Reaction conditions: NAC-800 catalyst (5.0 mg), corresponding solvent (1.50 mL), 230 °C, 48 h, hydrogen (20 bar).

In 2-PrOD-$d_1$

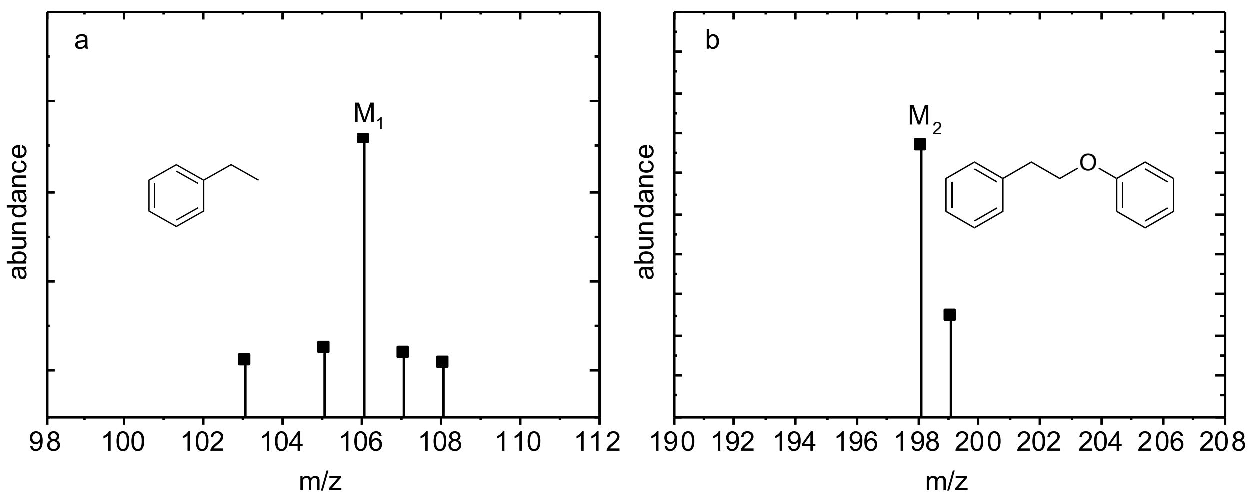


**Supplementary Fig. 29** Mass spectra of product (a) ethylbenzene and (b) PEB in 2-PrOH-$d_1$ over NAC-800 catalyst, showing no deuterium incorporation. Reaction conditions: NAC-800 (5.0 mg), 14 mmol $L^{-1}$ PPE in 2-PrOH-$d_1$ (1.50 mL), 230 °C, 48 h, $H_2$ (20 bar).

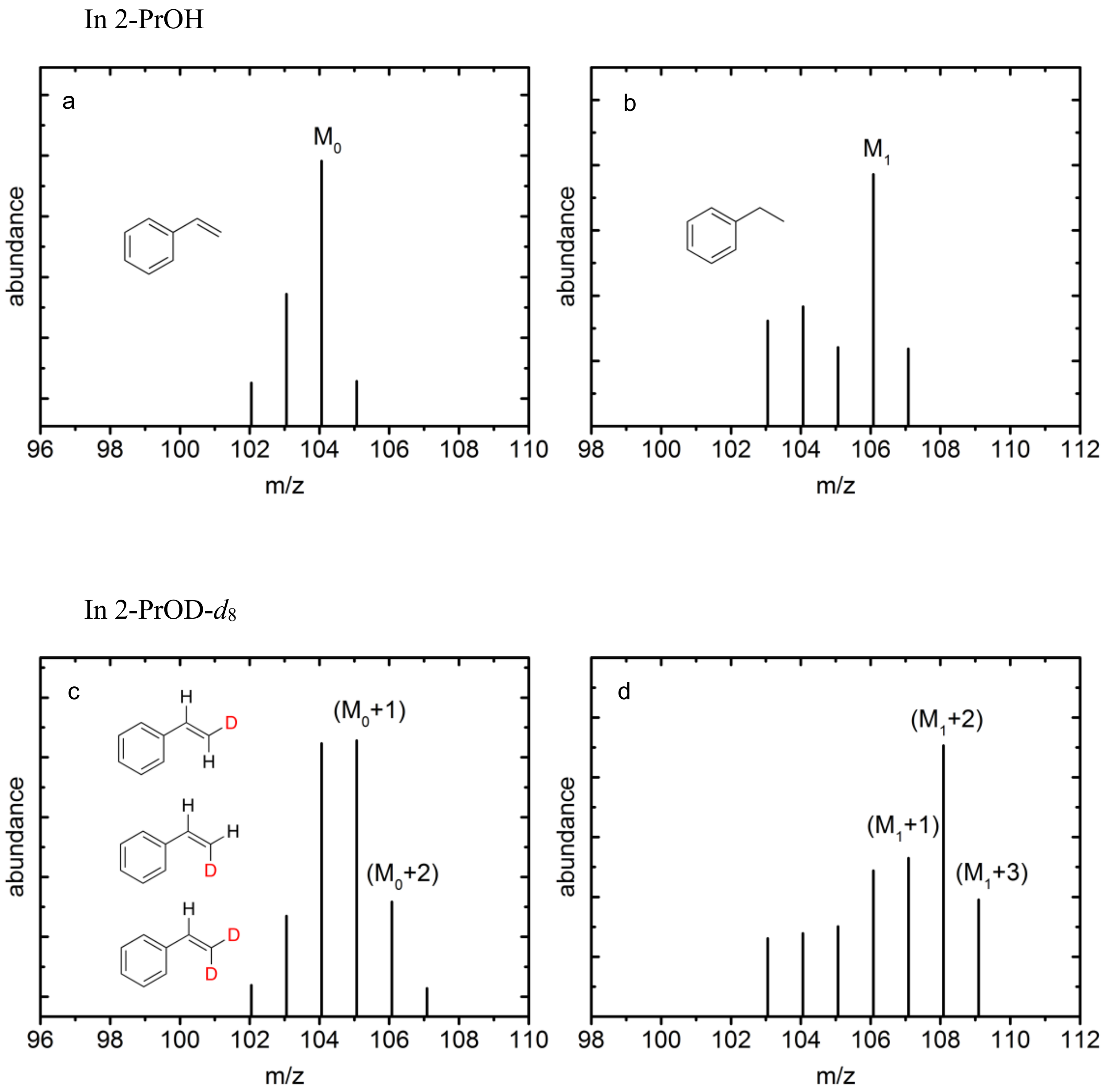


**Supplementary Fig. 30** Mass spectra of styrene and ethylbenzene in 2-PrOH (a and b) or 2-PrOH-$d_8$ (c and d) over NAC-800 catalyst. Reaction conditions: NAC-800 (5.0 mg), 56 mmol $L^{-1}$ styrene in corresponding solvent (1.50 mL), 230 °C, 4 h, $H_2$ (20 bar).

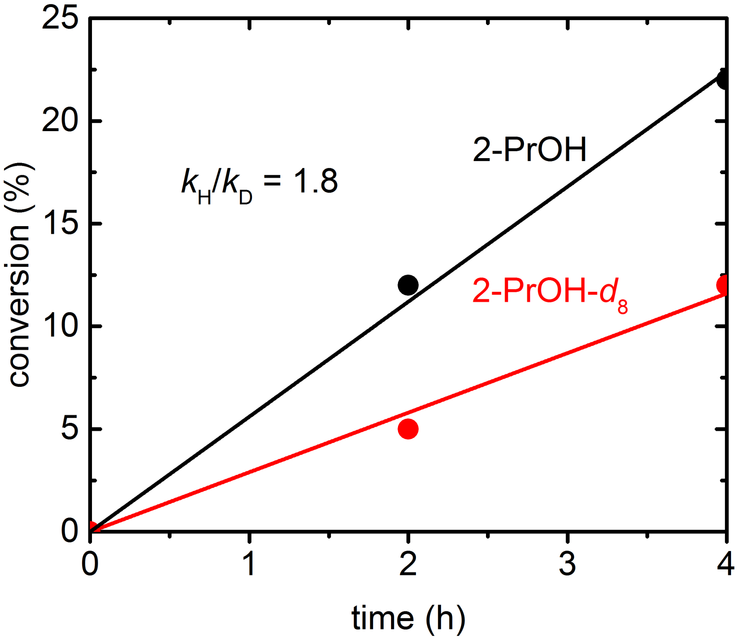


**Supplementary Fig. 31** Hydrogenation of styrene catalyzed by NAC-800. Reaction conditions: 56 mmol $L^{-1}$ styrene in 2-PrOH or 2-PrOH-$d_8$ (1.50 mL), NAC-800 (5.0 mg), 230 °C, 20 bar $H_2$.

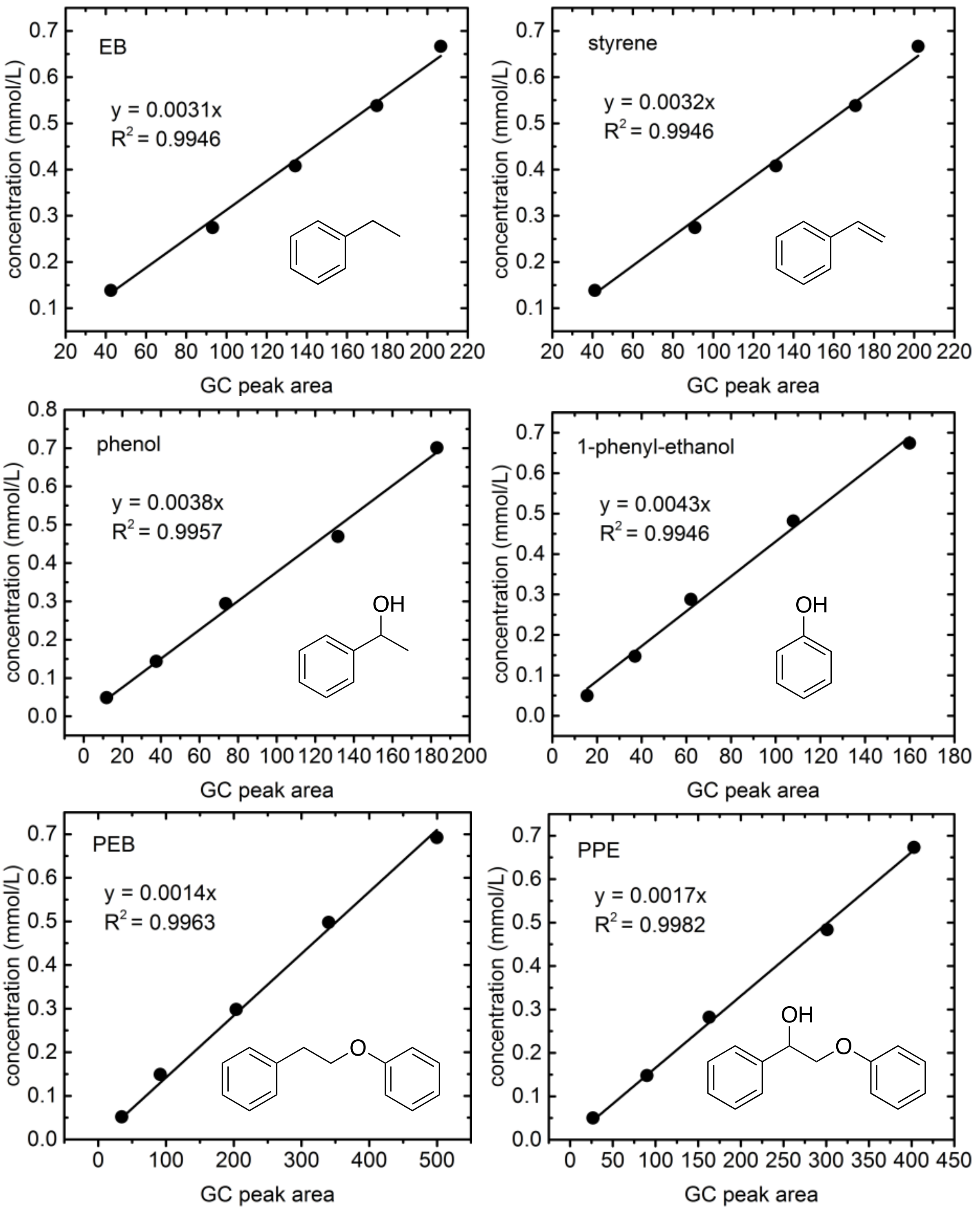


**Supplementary Fig. 32** Calibration curves of the molar concentration to the FID peak integration of key reaction species identified in PPE conversion catalyzed by NAC catalysts. These external calibrations with GC-FID were used to quantitate the concentrations of products and intermediates observed in PPE conversion.

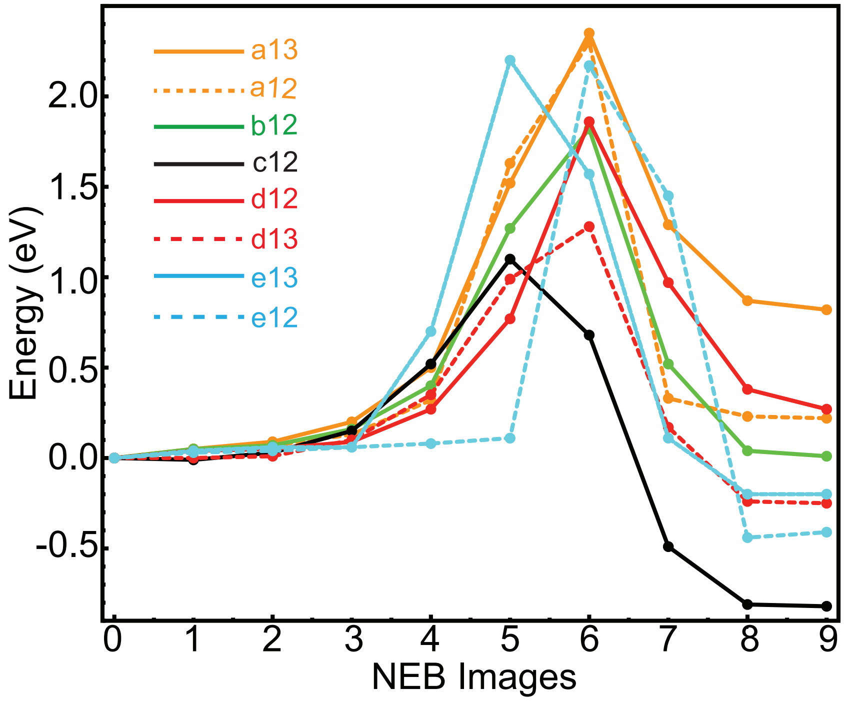


**Supplementary Fig. 33** Reaction profiles from DFT NEB calculations. Note these calculations were later used for further optimization to get the transition states using the Dimer method, so the values of the activation barrier from the NEB are very close to but not the same as the values reported in the main text, where the transition states were further optimized using the Dimer method and verified by calculating the vibrational frequencies.

**Supplementary Table 1** XPS and Elemental analysis of NACs. The error bars for CHNS are standard deviation (s.d.) from repeated measurements.

| entry | catalysts | XPS | | | CNHS | | |
|---|---|---|---|---|---|---|---|
| | | C(am%) | N(am%) | C:N (atomic) | C (wt%) | N(wt%) | C:N[b] (atomic) |
| 1 | NAC-600 | 75.56 | 19.76 | 3.8 | 66.94±0.17 | 21.66±0.01 | 3.6 |
| 2 | NAC-650 | 77.99 | 17.46 | 4.5 | 79.51±0.28 | 17.67±0.11 | 4.5 |
| 3 | NAC-700 | 79.29 | 15.00 | 5.3 | 71.36±0.31 | 16.92±0.06 | 4.9 |
| 4 | NAC-800 | 83.19 | 11.97 | 6.9 | 73.51±0.06 | 13.84±0.10 | 6.2 |
| 5 | NAC-900 | 85.72 | 9.00 | 9.5 | 77.93±0.32 | 9.87±0.02 | 9.2 |
| 6 | $N_{C3}$AC-800 | 88.79 | 5.82 | 15 | 77.82±0.19 | 6.80±0.01 | 13.0 |
| 7 | $N_{C4}$AC-800 | 86.74 | 5.26 | 16 | 79.79±0.32 | 7.15±0.01 | 13.0 |
| 8 | $N_mC_{glc}$-800 | 75.70 | 13.47 | 6.9 | 74.53±1.13 | 17.16±0.48 | 5.1 |
| 9 | $C_3N_4$ | 46.59 | 49.37 | 0.94 | 34.83±0.04 | 61.47±0.19 | 0.7 |
| 10 | NAC-800 after 7 cycles | 83.97 | 8.43 | 9.9 | 76.30±0.31 | 12.96±0.38 | 7.0 |

**Supplementary Table 2** N1*s* fitting parameters for NACs. The N1*s* peak of assigned N species are fitted with a narrow range for the binding energy according to theoretical calculation.[6]

| catalyst | pyridinic N | | pyrrolic N | | graphitic N | | pyridine N-oxide | |
|---|---|---|---|---|---|---|---|---|
| | position | FWHM | position | FWHM | position | FWHM | position | FWHM |
| NAC-600 | 398.3 | 2.0 | 400.1 | 1.7 | 401.2 | 1.7 | 403.4 | 2.0 |
| NAC-650 | 398.3 | 1.6 | 400.1 | 2.0 | 401.1 | 1.6 | 403.4 | 2.0 |
| NAC-700 | 398.3 | 1.7 | 400.1 | 1.8 | 401.0 | 2.0 | 403.4 | 2.0 |
| NAC-800 | 398.4 | 1.8 | 400.0 | 1.9 | 401.0 | 2.0 | 403.4 | 2.0 |
| NAC-900 | 398.4 | 1.7 | 400.0 | 1.6 | 401.1 | 1.9 | 403.3 | 1.8 |
| $N_{C3}$AC-800 | 398.4 | 1.8 | 400.1 | 1.5 | 401.0 | 2.0 | 403.4 | 1.9 |
| $N_{C4}$AC-800 | 398.4 | 1.7 | 400.1 | 1.6 | 401.0 | 2.0 | 403.4 | 1.6 |

**Supplementary Table 3** N1*s* fitting data of NACs. The error bars are estimated based on the uncertainties of N1*s* XPS deconvolution.

| entry | catalysts | % of total N1*s* | | | |
|---|---|---|---|---|---|
| | | pyridinic N | pyrrolic N | graphitic N | pyridine N-oxide |
| 1 | NAC-600 | 61.1±0.4 | 27.0±0.7 | 8.0±0.6 | 3.9±0.2 |
| 2 | NAC-650 | 41.1±0.4 | 36.5±1.5 | 17.9±0.7 | 4.5±0.1 |
| 3 | NAC-700 | 44.3±0.2 | 26.3±0.1 | 24.6±0.2 | 4.7±0.1 |
| 4 | NAC-800 | 40.8±0.3 | 12.9±0.1 | 40.1±0.4 | 6.2±0.4 |
| 5 | NAC-900 | 35.5±0.2 | 10.7±0.1 | 48.0±0.5 | 5.7±0.2 |
| 6 | $N_{C3}$AC-800 | 35.2±0.4 | 10.1±0.2 | 49.1±2.4 | 5.6±0.3 |
| 7 | $N_{C4}$AC-800 | 34.2±0.5 | 10.8±0.6 | 49.9±3.6 | 5.0±0.2 |
| 8 | NAC-800 after 7 cycles | 39.9±0.3 | 17.1±0.1 | 38.6±0.5 | 4.3±0.2 |

**Supplementary Table 4** Catalytic conversion of PPE on different NACs. Two types of carbon tetrachloride and two types of ethylenediamine from different vendors were used for the synthesis of NACs. The resulting three NAC-800 catalysts show nearly the same activity for PPE conversion.

| entry | carbon tetrachloride | ethylenediamine | conversion (%)[a] | | | |
|---|---|---|---|---|---|---|
| | | | test 1 | test 2 | test 3 | average |
| 1 | ≥99.5%, Sigma-Aldrich | ≥99.5%, Sigma-Aldrich | 28 | 29 | 30 | 29 |
| 2 | ≥99.5%, Sigma-Aldrich | 99%, Acros Organics | 29 | 28 | 28 | 28 |
| 3 | ≥99.9%, Sigma-Aldrich | ≥99.5%, Sigma-Aldrich | 27 | 28 | 28 | 28 |

[a] Reaction conditions: catalyst (5.0 mg), PPE (14 mmol $L^{-1}$), 2-PrOH (1.50 mL), $H_2$ (20 bar), 230 °C, 8 h.

**Supplementary Table 5** Conversion, product yield and carbon balance for PPE conversion by various NAC catalysts, in Fig. 2b.

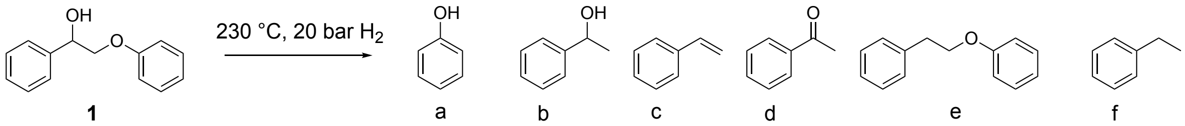


| exp | catalysts | conversion (%) | yield (%) | | | | | | carbon balance (%) | |
|---|---|---|---|---|---|---|---|---|---|---|
| | | | a | b | c | d | e | f | C6 | C8 |
| 1 | NAC-600 | 0 | 0 | 0 | 0 | 0 | 0 | 0 | - | - |
| 2 | NAC-650 | 7 | 4 | 1 | 2 | 0 | 2 | 2 | 99 | 99 |
| 3 | NAC-700 | 14 | 11 | 2 | 2 | 0 | 3 | 8 | 100 | 101 |
| 4 | NAC-800 | 28 | 24 | 4 | 7 | 0 | 5 | 11 | 101 | 99 |
| 5 | NAC-900 | 25 | 19 | 5 | 6 | 0 | 6 | 9 | 101 | 101 |

**Supplementary Table 6** PPE conversion by various control catalysts. Reaction conditions: control catalyst, 14 mmol $L^{-1}$ PPE in 2-PrOH (1.50 mL), $H_2$ (20 bar), 230 °C, 4 h.

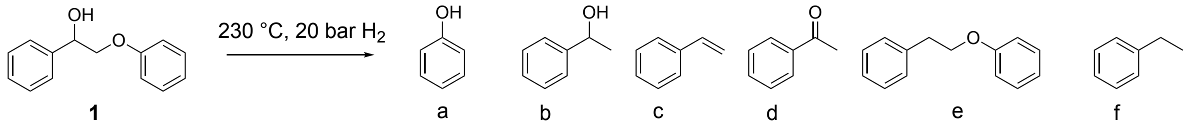


| exp | catalysts | conversion (%) | yield (%) | | | | | | carbon balance (%) | |
|---|---|---|---|---|---|---|---|---|---|---|
| | | | a | b | c | d | e | f | C6 | C8 |
| 1 | NAC-800 | 19 | 16 | 3 | 13 | 0 | 3 | 0 | 100 | 100 |
| 2 | $FeCl_3$[a] | 0 | - | - | - | - | - | - | - | - |
| 3 | $CuCl_2$[a] | 0 | - | - | - | - | - | - | - | - |
| 4 | $NiCl_2$[a] | 0 | - | - | - | - | - | - | - | - |
| 5 | Fe/NAC-800-(800Ar)[b] | 14 | 9 | 1 | 0 | 0 | 5 | 7 | 100 | 99 |
| 6 | Fe/NAC-800-(500$H_2$)[c] | 19 | 14 | 3 | 1 | 0 | 5 | 9 | 100 | 99 |

[a] 0.105 mmol; [b] 5.0 mg, Fe loading is 7.5 wt%; [c] 5.0 mg, Fe loading is 0.7 wt%.

**Supplementary Table 7** Calculated TOF values of NAC-800 and other reference metal catalysts.

| catalyst | reactant | temperature (°C) | $H_2$ (bar) | time (h) | Conversion (%) | TOF ($h^{-1}$) | Reference |
|---|---|---|---|---|---|---|---|
| NAC-800 [a] | **1**, PPE | 230 | 20 | 2 | 8.9 | 32 | This work |
| NAC-800 [a] | **5**, BPE | 230 | 20 | 2 | 17 | 61 | This work |
| $N_mC_{glc}$-800 | **1**, PPE | 230 | 20 | 8 | 0 | 0 | This work |
| $C_3N_4$ | **1**, PPE | 230 | 20 | 8 | 0 | 0 | This work |
| 57 wt% $Ni/SiO_2$ | PEB | 120 | 6 | 1.5 | 8.1 | 20 | *J. Am. Chem. Soc.* **134**, 20768-20775 (2012) |
| 5 wt% Ru/C | **1**, PPE | 150 | -- [b] | 10 | 66.7 | 1.3 | *ACS Sus. Chem. Eng.* **6**, 2872-2877 (2018) |
| 56 wt% $Ni/Al_2O_3$ | **5**, BPE | 130 | -- [b] | - | 15.4 | 0.27 | *Chem. Sci.* **10**, 4458-4468 (2019) |

[a] the density of active sites is assumed to be the same as the hydrogen uptake measured and calculated by pulsed chemisorption at 240 °C. [b] using 2-PrOH as hydrogen source.

**Supplementary Table 8** B.E.T. surface area and pore parameters of NACs.

| entry | samples | $S_{B.E.T.}$ ($m^2$ $g^{-1}$) | $V_{pore}$ ($cm^3$ $g^{-1}$) | $D_p$ (nm) |
|---|---|---|---|---|
| 1 | NAC-600 | 583 | 0.68 | 6.1 |
| 2 | NAC-650 | 700 | 0.63 | 3.6 |
| 3 | NAC-700 | 765 | 0.78 | 4.8 |
| 4 | NAC-800 | 648 | 0.67 | 4.4 |
| 5 | NAC-900 | 788 | 0.60 | 4.9 |
| 6 | $N_{C3}$AC-800 | 582 | 0.75 | 4.7 |
| 7 | $N_{C4}$AC-800 | 562 | 0.68 | 4.2 |
| 8 | NAC-800 after 7 cycles | 711 | 0.54 | 5.2 |

**Supplementary Table 9** C1*s* fitting data of different NACs. The C-C/C=C percentage was increased by increasing calcination temperature due to the loss of N, while the content of C=N is maximal at 800 °C.

| entry | catalysts | C-C/C=C | C-N | C=N |
|---|---|---|---|---|
| 1 | NAC-600 | 31.2 | 48.1 | 20.7 |
| 2 | NAC-650 | 33.9 | 44.6 | 21.6 |
| 3 | NAC-700 | 37.4 | 39.2 | 23.3 |
| 4 | NAC-800 | 37.2 | 38.3 | 24.5 |
| 5 | NAC-900 | 39.4 | 38.0 | 22.6 |
| 6 | $N_{C3}$AC-800 | 38.4 | 38.6 | 23.0 |
| 7 | $N_{C4}$AC-800 | 41.5 | 37.7 | 20.8 |
| 8 | NAC-800 after 7 cycles | 35.9 | 40.0 | 24.1 |

**Supplementary Table 10** Metal-free dehydrogenation of THQ or hydrogenation of quinoline over NAC-800 catalyst.

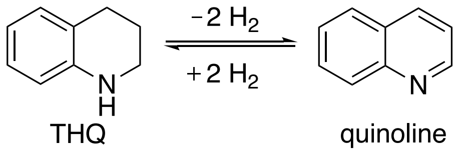


| entry | reactant | equilibrium shift to | temperature (°C) | gas | pressure (bar) | conversion (%) |
|---|---|---|---|---|---|---|
| 1 | THQ | dehydrogenation | 140 | $H_2$ | 10 | 6 |
| 2 | quinoline | hydrogenation | 140 | $H_2$ | 10 | 5 |
| 3 | THQ | dehydrogenation | 230 | $H_2$ | 20 | 10 |
| 4 | quinoline | hydrogenation | 230 | $H_2$ | 20 | <1 |

**Supplementary Table 11** PPE conversion by various NACs and control catalysts.[a]

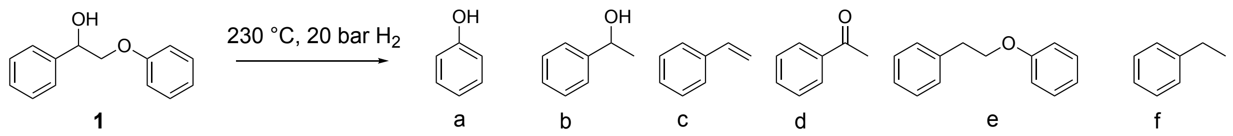


| exp | catalysts | conversion (%) | yield (%) | | | | | | carbon balance (%) | |
|---|---|---|---|---|---|---|---|---|---|---|
| | | | a | b | c | d | e | f | C6 | C8 |
| 1 | - | 0 | - | - | - | - | - | - | - | - |
| 2 | $N_{C3}$AC-800 | 12 | 9 | 3 | 2 | 1 | 4 | 4 | 100 | 101 |
| 3 | $N_{C4}$AC-800 | 2 | 2 | <1 | <1 | 0 | <1 | 1 | 100 | 100 |
| 4 | Cabot carbon black | 0 | - | - | - | - | - | - | - | - |
| 5 | $N_mC_{glc}$-800 | 0 | - | - | - | - | - | - | - | - |
| 6 | $C_3N_4$ | 0 | - | - | - | - | - | - | - | - |

[a] Reaction conditions: catalyst (5.0 mg), 14 mmol $L^{-1}$ PPE in 2-PrOH (1.50 mL), $H_2$ (20 bar), 230 °C, 8 h.

**Supplementary Table 12** DFT-calculated imaginary frequencies of the transition states.

| Configurations | Imaginary frequencies (THz) |
|---|---|
| a12 | 31.3 |
| a13 | 54.9 |
| b12 | 47.5 |
| c12 | 38.5 |
| d12 | 48.0 |
| d13 | 25.3 |
| e12 | 43.3 |
| e13 | 24.8 |

## Supplementary References


S1 Galkin, M. V. & Samec, J. S. Selective Route to 2‐Propenyl Aryls Directly from Wood by a Tandem Organosolv and Palladium‐Catalysed Transfer Hydrogenolysis. *ChemSusChem* **7**, 2154-2158 (2014).

S2 Luo, J., Zhang, X., Lu, J. & Zhang, J. Fine Tuning the Redox Potentials of Carbazolic Porous Organic Frameworks for Visible-Light Photoredox Catalytic Degradation of Lignin β-O-4 Models. *ACS Catal.* **7**, 5062-5070 (2017).

S3 Huo, J. *et al.* Improved hydrothermal stability of Pd nanoparticles on nitrogen-doped carbon supports. *Catal. Sci. Technol.* **8**, 3548-3561 (2018).

S4 Talapaneni, S. N. *et al.* Facile synthesis and basic catalytic application of 3D mesoporous carbon nitride with a controllable bimodal distribution. *J. Mater. Chem.* **22**, 9831-9840 (2012).

S5 Johnson, K., Purvis, G., Lopez-Capel, E. *et al.* Towards a mechanistic understanding of carbon stabilization in manganese oxides. *Nat. Commun.* **6**, 7628 (2015).

S6 Ayiania, M. *et al.* Deconvoluting the XPS spectra for nitrogen-doped chars: An analysis from first principles. *Carbon*, **162**, 528-544 (2020).
